# Assessing and updating the environmental and conservation status of three endangered endemic plants in light of potential climate change in Saint Catherine Protectorate, Egypt

**DOI:** 10.1186/s12870-025-07127-z

**Published:** 2025-09-16

**Authors:** Mohamed M. El-Khalafy, Asmaa S. Abo Hatab, Yassin M. Al-Sodany, Kamal H. Shaltout, Heba M. Bedair

**Affiliations:** 1 Botany and Microbiology Department, Faculty of Science, Kafrelsheikh University, Kafrelsheikh, 33511 Egypt; 2 Botany and Microbiology Department, Faculty of Science, Tanta University, Tanta, 31527 Egypt

**Keywords:** Prediction, Conservation, IUCN red list assessment, Endemism, Extinction, Saint Catherine Protectorate, Habitat suitability.

## Abstract

**Background:**

Evaluating the effect of climate change on the distribution patterns of endemic plants in the mountainous areas is critical for understanding the dynamics of this terrestrial ecosystem in the uncertainty of future changes. The population size of the endemic plants in Saint Catherine protectorate (SKP) has declined significantly over the previous century, as the climatic changes, especially drought and high temperature are the most threats that influenced the presence of them.

**Results:**

Three endangered endemic taxa (*Anarrhinum forskaohlii* subsp. *pubescens*, *Origanum syriacum* subsp. *sinaicum*, and *Polygala sinaica* var. *sinaica*) were assessed depending on IUCN categories and criteria. Besides, the response of these taxa to anticipated climate change over the next few decades was investigated using species distribution models (SDMs). Our analysis included insertion of bioclimatic and soil variables in the SDM modeling process and incorporation of four main algorithms (generalized linear model (GLM), Random Forest (RF), Boosted Regression Trees (BRT), and Support Vector Machines (SVM) in an ensemble model. RF and GLM algorithms outperformed the other algorithms, underscoring their efficacy in predicting the distribution of endemics in Saint Catherine Protectorate (SKP). An examination of the relative significance of bioclimatic variables revealed that wind and climate variables were dominant in shaping the potential distribution of the three taxa. Wind, Bio9, Bio3, Vol. water content at -10kpa (water10) and elevation were the most effective variables for *A. forskaohlii* subsp. *pubescens*; wind, Bio3, Bio15, clay, and elevation for *O. syriacum* subsp. *sinaicum*; wind, Bio3, Bio8, clay, aridity index and elevation for *P. sinaica* var. *sinaica*. In addition, our projections showed that the potential range of *O. syriacum* subsp. *sinaicum* is expected to decrease over the next few decades under both scenarios SSP585 and SSP126. On the other hand, *A. forskaohlii subsp. pubescens* and *P. sinaica* var. *sinaica* are expected to expand over the next few decades under both scenarios.

**Conclusions:**

Controlling the risk that threaten these species and implementing protection measures is essential. It is crucial to enact laws and regulations to ensure their safeguarding. Our discoveries highlight the urgency of conservation measures such as reintroduction, in situ and *ex situ* conservation planning in appropriate habitats.

## Introduction

Species extinction is one of the urgent global issues laid at the basis of biodiversity conservation [1]. The decline of wildlife is a global crisis with extinction rates up to 1000 times greater than historical levels and future rates that are projected to increase [2]. At the global scale, about 40,000 species are now evaluated as threatened according to IUCN categories [3]and up to 1 million species are at risk of extinction in the next several decades [4].

Current research demonstrates that climate change is already affecting biodiversity, with impacts expected to worsen significantly without effective mitigation [5]. Studies reveal uneven extinction risks among species and regions, even under minimal warming [6], driving varied global responses. Despite extensive research on climate change effects, key gaps remain in understanding risk patterns across ecological scales (species to communities), ecosystems (terrestrial, freshwater, marine), endemicity levels, and emission scenarios. Systematic analysis of these factors is crucial for improving risk assessment, guiding conservation priorities, and shaping adaptation strategies. Species are responding to climate change primarily through geographic range shifts, expansions, and contractions [7]. Broad-ranging species generally face lower vulnerability, as portions of their distribution may act as climatic refugia [8], while invasive species often demonstrate resilience due to their adaptive capacity [9]. In contrast, endemic species face heightened extinction risks from climate-exacerbated threats like habitat loss and invasive species interactions, compounded by their small ranges, niche specialization, limited dispersal, and low adaptive potential [10, 11]. Consequently, regions rich in endemics exhibit disproportionate vulnerability at both species and ecosystem levels [12, 13].

Anthropogenic climate change has been altering global conditions since the Industrial Revolution, with projections indicating global mean temperatures will likely rise by at least 1.5 °C within the next two decades. This warming is driving significant shifts in precipitation patterns, wind dynamics, and cryospheric conditions worldwide [5]. Concurrently, the dual pressures of climate change and land-use change over the past two centuries have triggered widespread species extinctions, biodiversity decline, and ecological homogenization across ecosystems [14]. Notably, land-use change currently represents the most severe threat to global biodiversity [15]. These two drivers (climate change and land-use/land-cover (LULC) change) often interact synergistically, exacerbating their individual impacts on species and ecosystems [16].

Despite tremendous efforts over the past decades to conserve biodiversity, it still poses significant challenges and appears to be lost when it come at the global level [14]. For more than 50 years, there have been international initiatives to identify and conserve threatened wildlife starting with the first International Union for Conservation of Nature (IUCN) list of threatened species published in 1964 [17]. Numerous plant resources of the spontaneous flora are in danger of extinction as a result of uncontrolled harvesting, improper agricultural and forestry practices, urbanization, pollution, habitat destruction, and ecological fragmentation, as well as factors other than anthropogenic pressure (such as climate changes, ecological collapse, and competition with non-native invasive species [18, 19].

Although the current extinction rates are nearly known, extinction quantification remains critically important for improving the accuracy of extinction estimates and prediction [20]. The measurement of in situ conservation of endemic and threatened plant species is an effective tool to protect them against extinction [21]. The IUCN Red List includes various endemic species that are at risk of global extinction due to their limited geographical range and very specific habitats [22, 23]. Therefore, it is crucial to safeguard and preserve these species by assessing the potential distribution of suitable habitats and determining the environmental factors that influence their presence and survival in both current and future conditions [23–25]. Identifying the current geographic distribution, population status, and threats that expose these taxa to the risk of extinction is the initial step in starting conservation processes for them [21].

An endemic species is limited to a specific geographic region due to factors such as isolation or in response to abiotic environments. Understanding endemicity is crucial for determining conservation priorities [26]. The restricted geographical range of the endemic taxa generally indicates greater vulnerability than other taxa, so it*’*s used as a surrogate to identify the conservation priorities [27]. Different numbers of endemic taxa in Egypt are provided by previous literature: e.g., 69 taxa [28], 60 taxa [29], 76 taxa [30], 48 taxa [31] and 41 taxa [32].The distribution of taxa in many countries is poorly understood [33, 34], because of biased species collection, inadequate sampling techniques, limited research resources and facilities, and challenges in species identification and definition [35]. Extinction is a severe threat that impacted over 39% of the estimated plant species in the world [36]. The sixth wave of extinction has been observed by numerous researchers around the world [35, 37–39], as plant diversity is threatened with an unprecedented diversity of habitats, species, and genetic levels. One of the most powerful indicators of the likelihood of extinction in land-based species is their limited geographical range [10, 40].

Over the past few years, numerous countries have invested significant resources into producing the IUCN Red Lists in accordance with the IUCN protocol [41, 42]. The IUCN Red List categories and criteria are widely recognized as the most comprehensive and authoritative method available for evaluating the global risk of species extinction [43]. Red List data, which includes habitat criteria, threats, and conservation measures, can be utilized to pinpoint and aid in the crafting of conservation and restoration strategic plans for species that necessitate special conservation efforts [17]. The IUCN red list methodology employs criteria based on population size, rate and potential causes of decline, and distribution area to allocate species to categories reflecting their relative risk of extinction [43].

The ecological niche of a species is the interaction between the space and the conditions in which it can survive, persist, and maintain its ability to reproduce and sustain a viable population [44]. The primary factors influencing the distribution and ecological niche of species at different geographic scales are climate, soil characteristics, topography, land utilization, and biological interrelations [45]. Global warming has the potential to alter the distribution of natural species, particularly those with limited geographic range or endemic species that struggle to adapt to changing climatic conditions, putting them at risk of endangerment or extinction [46, 47]. Species distribution models (SDMs) or ecological niche models are tools used to predict suitable habitats for specific species by analyzing the environmental factors within their natural habitats [48]. In addition, SDM can guide conservationists in predicting the effects of global warming, land use change, discovering unsuitable areas as well as suitable high presence areas to further investigation, reintroduction or natural conservation of these endangered species [49–51]. SDMs are mainly responsible for understanding the impact of the environment on the distribution of a species in its natural habitat. To accomplish this, an SDM is created by gathering data on species presence and environmental characteristics (including climate and topography) stored in a geographic information system. The numerical results of statistical SDMs are often simplified into environmental suitability indices, which range from 0 (unsuitable) to 1 (optimal). Additionally, it has been demonstrated that this index is frequently connected not only to the likelihood of occurrence but also to other crucial population parameters, such as growth rate, surface area, and the number of vegetative and reproductive individuals [52, 53].

The accuracy and predictive capability of any species distribution model are determined by the reliability of the field data used and the environmental variables chosen for inclusion in the model [54]. The availability of high spatial resolution environmental predictors is critical for modeling species distribution at landscape scales [55]. Obtaining high-resolution environmental data in unexplored areas poses a significant challenge. A major concern for researchers is the lack of geographical resources (e.g., topographic and climatic databases) that accurately represent developing countries and impoverished regions. It’s important to acknowledge that despite their high resolution, maps may still contain commission errors due to limitations in the availability and resolution of the predictor variables used, impacting the accuracy and precision of species distribution models [56]. Previous efforts to reduce omission errors in coarse resolution maps also resulted in decreased underestimation of species habitat suitability, highlighting diversity hotspots in areas that were previously overlooked in earlier maps [57].

Endemic species play a crucial role in Egypt’s plant life. Many of these species are at a high risk of extinction due to significant threats such as overcollection, overgrazing, and construction activities [32]. Hence, this research seeks to enhance the assessment of three endemic species in SKP by: 1- conducting IUCN evaluations of these endemic species, including an analysis of their ecological and conservation status, as well as their level of risk and change in status over previous years; 2- proposing new assessments and comparing them with previous assessments; 3- utilizing species distribution modeling methods to estimate the impact of environmental changes on their distribution; 4- predicting the potential distribution of these native species; 5- identifying the environmental factors that influence their distribution; and 6- evaluating the potential changes in their geographical range under different climate change scenarios.

## Materials and methods

### Study area and climate change

**Fig. 1:**
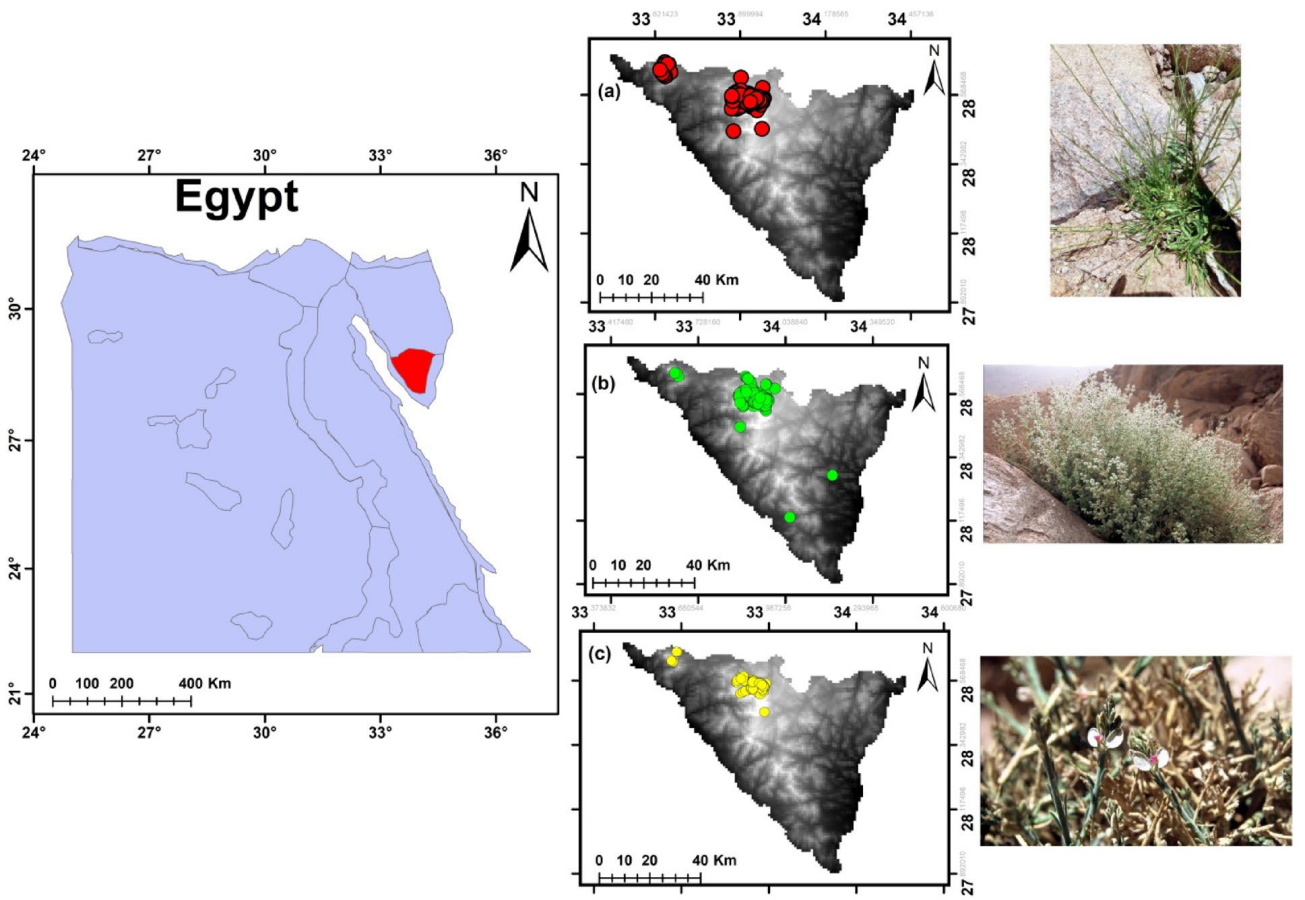
Map of the study area showing SKP region indicating locations of the collected occurrence records of (**a**) *Anarrhinum forskaohlii* subsp. *pubescens*, (**b**) *Origanum syriacum* subsp. *sinaicum*, and (**c**) and *Polygala sinaica* var. *sinaica*

Sinai is a characteristic triangular-shaped region and is bound by water bodies along long stretches in all directions. It possesses its own characteristics. It is bound from the north by the Mediterranean Sea, from the west by the Gulf and Isthmus of Suez, and from the east by the Gulf of Aqaba and the Palestinian-Israeli boundary. Sinai covers about 6% (61,000 km^2^) of the total area of Egypt. Sinai’s coasts extend for approximately 700 km, making it less continental than other regions of Egypt. Sinai represents nearly all of Egypt’s geologic formations, structures, and landforms. Additionally, the climatic variations in Sinai closely resemble those found in Egypt. The southern part of Sinai is characterized by mountains, while the central part is a tableland area. The northern region is divided into two sections: the southern portion is characterized by solitary domal hills and mountains, while the northern section is predominantly blanketed by sand dunes [58] (Fig. 1). The unique topography of SKP includes gorges, slopes, terraces, caves, and ridges, each of which provides a habitat for specific plant communities [59, 60]. SKP experiences a wide range of air temperatures and levels of precipitation. It is known as the coolest region in Egypt and the only one where snowfall occurs [60]. The average monthly temperatures vary from 8.6 °C in January to 25.5 °C in August. The average annual rainfall from 1970 to 2017 was minimal and irregular, at 37.5 mm, but sudden and unpredictable flash floods with up to 300 mm of rainfall have been recorded (in 2012 and 2014) [60]. Between 1979 and 1992, the coldest monthly mean minimum temperatures occurred in January and February, dropping to 1.4 °C, while the warmest mean maximum temperatures were recorded in June and July, reaching 30.8 °C and 31.8 °C, respectively [61].

South Sinai experiences an arid to hyper-arid climate, marked by prolonged, hot, and dry summers alongside mild winters. The region’s significant altitudinal variations contribute to considerable fluctuations in air temperature across different areas. Climate data from Saint Catherine in Sinai, Egypt (2010–till 2020), reveal striking evidence of rapid climatic shifts, including declining precipitation and rising average temperatures. If this trend persists, prolonged droughts are likely to intensify, disrupting plant growth and altering species distribution patterns. Such changes could detrimentally influence the population dynamics of numerous plant and animal species that are critical to local communities, and this cause potentially leading to ecological and socioeconomic consequences [60, 62]. Ecosystems and biodiversity face growing threats from intensifying climate change. Rising temperatures and shifting climate patterns directly affect species survival, alter ecological relationships, and transform habitats, ultimately disrupting ecosystem functions and reducing nature’s capacity to provide essential goods and services for human societies [63].

**Mountain ecosystems** are shaped by dynamic interactions between regional climate and local geophysical factors, creating diverse microhabitats [64]. In SKP, this interplay of varied landforms, geological structures, and climatic heterogeneity has led to the formation of six distinct microhabitat types, as classified by Khedr [65]: wadis, terraces, slopes, gorges, farshes (basins), and caves, each supporting unique environmental conditions and specialized plant communities [65]. Due to this ecological complexity, St. Catherine PA is recognized as one of the most floristically rich hotspots in Egypt, hosting a remarkable diversity of medicinal, rare, and endemic plant species. Notably, the area harbors 13 endemic vascular plants, representing approximately 30% of Egypt’s total endemic flora

###  Description of the target species

*A. forskaohlii* subsp. *pubescens* is a perennial herb that grows exclusively in mountainous regions, particularly within granite-based wadis, gorges, and steep slopes. It thrives on sharply inclined terrain, with slopes reaching up to 90°, and shows a preference for north-facing (26%), west-facing (24%), northeast-facing (16%), and northwest-facing (16%) exposures. It has a narrow altitudinal range between 1000 and 2400 m [66]. Its soil varies by location: gravelly in wadis and plains, rocky on mountain surfaces, and sandy to loamy sand in texture. These soils are alkaline, ranging from non-saline to slightly saline, and exhibit low levels of essential nutrients and cation-exchange capacity (CEC) [67]. *A. forskaohlii* subsp. *pubescens* is a perennial plant covered with glandular hairs. It has upright, wiry stems that branch from the base. The basal leaves are oblanceolate with coarse teeth in the upper portion, tapering to a pointed tip, and narrowing into a short petiole (3–8 mm long). The stem leaves lack petioles (sessile), are narrow-elliptic to linear, smooth-edged (entire), and pointed. The plant blooms in elongated, spike-like racemes. Its bracts are slender (filiform), measuring 2–3 mm, while the calyx is 1–1.2 mm long, split into five lance-shaped lobes with thin, dry (scarious) margins. The small white corolla is 3–3.5 mm long. The fruit is a round (globose), hairless capsule about 2.5 mm in size [68]. Flowering occurs in late spring, with seed production and dispersal taking place by late summer.

*Origanum syriacum* subsp. *sinaicum* is a perennial, aromatic herb with a woody base and erect or ascending, branched, pubescent stems. Its leaves are small (5–15 mm long, 3–8 mm wide), ovate to elliptic, with entire or slightly toothed margins, and densely tomentose, especially beneath. The inflorescence consists of dense, spike-like clusters of white or pale pink bilabiate flowers (~ 5 mm long), each with a tubular, hairy calyx (~ 3 mm) and four exserted stamens. The bracts are ovate, overlapping, and green to purplish. The fruit forms four smooth, brown nutlets (~ 1 mm long). Flowering occurs in summer (June–August), and the subspecies typically grows in rocky, arid habitats of the Sinai Peninsula [68]. It primarily inhabits rocky, arid mountain slopes and wadis in the Sinai Peninsula, particularly in limestone-rich, well-drained substrates at elevations between 1,200 and 2,200 m [68]. It favors sun-exposed, xeric microhabitats such as rock crevices, gravelly terraces, and stony ridges, where water runoff prevents prolonged soil moisture.

*Polygala sinaica* is a perennial herb with a distinct life cycle. Seedlings emerge between March 1 st and May 1 st, transitioning into the early vegetative stage. By late spring, the plant reaches the late vegetative and flowering stages. Fruit maturation occurs in summer, after which the plant dries out, completing its life cycle. Seeds remain dormant in the soil until the following spring, when they germinate to produce new individuals. *Polygala sinaica* predominantly occurs on steep granite cliffs (65% of recorded sites) and montane gorges (30%). This species is exclusively found in high-elevation wadis featuring near-vertical slopes (≤ 90° inclination). Slope aspect analysis reveals preferential establishment on western (30%), northeastern (15.5%), southwestern (15.5%), and northwestern (7%) exposures. The species demonstrates optimal occurrence frequencies at elevations ranging from 1600 to 2200 m above sea level [69]. *Polygala sinaica* var. *sinaica*, also known as Sinai milkwort, is a perennial, shrubby plant with alternate leaves and pink or white flowers. It has a robust, woody stem and exhibits a distinctive “papilionaceous” flower structure, with two prominent, winged sepals. Inflorescence is terminal or axillary racemes (flower clusters). The flowers are bisexual and zygomorphic, meaning five they have distinct upper and lower parts. Sepals are 5, unequal, with two large, petaloid (petal-like) inner sepals. Petals are three, connate (joined together) at the base, forming a distinctive keel. Stamens are eight, with filaments united at the base, forming a staminal sheath. Ovary is 2-loculed (two chambers), with one ovule per locule. Fruit is capsular, compressed, and often winged. Seeds are homogeneous, with a range of colors from light brown to black [68].

### Field excursions and distribution data

The species’ occurrence data were gathered from multiple sources: a- Field surveys conducted between 2016 and 2023 (The majority of the field trips were conducted in Spring season), b- herbarium collections at Tanta University (TANE), Alexandria University (ALEX), Cairo University (CAI), Assiut University (ASTU), Agricultural Research Center (CAIM), Desert Research Center (CAIH), National Research Centre (CAIRC), and Kafrelsheikh University (KFSUH), c- National Registry for Egyptian Herbaria (accessed on 8 September 2024), and d- The Global Biodiversity Information Facility (GBIF) (accessed on 14 October 2024). Additionally, field visits were carried out to record habitat types, geographic coordinates, and threats. Specimens were collected from different sites during these surveys. On the other type, data processing involved: a- imputing missing values using the “missForest” package, b- manipulating data with “dplyr” (v1.1.3), c- structuring datasets with “tidyr” (v1.2.0), and d- managing spatial data and filtering outliers using the “raster” package in R 4.3.1.

Species identification and synonyms were verified using published references [28, 68] and Plants of the World Online (POWO). Plant specimens were collected in compliance with national regulations and international guidelines (IUCN and CITES). Authorization for scientific collection was granted by the Department of Botany and Microbiology, Faculty of Science, Kafrelsheikh University. Voucher specimens (deposition numbers 2117–2124) were preserved in the KFUH Herbarium at Kafrelsheikh University. Taxonomic identification was conducted by Prof. Yassin M. Al-Sodany (Plant Ecology and Flora, Kafrelsheikh University) and Prof. Selim Z. Heneidy (Plant Ecology and Flora, Alexandria University).

### IUCN red list assessment

IUCN Red List Categories and Criteria: Version 3.1 was utilized to assess the current ecological and conservation conditions of these plants [70], and its guidelines [43]. The evaluation process involved comparing the assessment previously conducted with the updated List based on the current study. Criterion B, which pertains to the geographic range as either B1 (extent of occurrence) AND/OR B2 (area of occupancy), is utilized to collect the following data:

#### Geographic range, characteristics of the population and its habitat

In ArcGIS 10.4.1, we plotted the study locations (Fig. 1). We evaluated the Extent of Occurrence (EOO), the Area of Occupancy (AOO), and the number of locations where the species are present using the Eoo calculator in ArcGIS and red package in R [10]. The target species’ occurrence data was quantified according to IUCN Standards and Petitions Committee guidelines, including: total number of occupied locations, EOO and AOO. Geographic coordinates were recorded in decimal degrees (WGS 84 datum) with a Garmin eTrex 30 GPS receiver, achieving five-decimal-place precision. Elevation data were collected in meters above sea level. All spatial data were mapped using ArcGIS 10.4.1 software.

.The present study detected the phytogeographic regions according to Boulos [29]. To determine the number of subpopulations and populations of the target species, the total count of all individuals and mature individuals was conducted through filed trips. The total number of individuals and the number of mature individuals were recorded to determine the size of the subpopulations and the overall population of the studied species. Population size and the number of mature individuals were estimated on the basis of data accuracy and uncertainty levels presented in the IUCN Standards and Petitions Committee (2019) standards. In addition, the habitats of the target species were detected according to IUCN Habitats Classification Scheme ver. 3.1 (https://www.iucnredlist.org/resources/classificationschemes).

#### Threats and red list categories

**Fig. 2:**
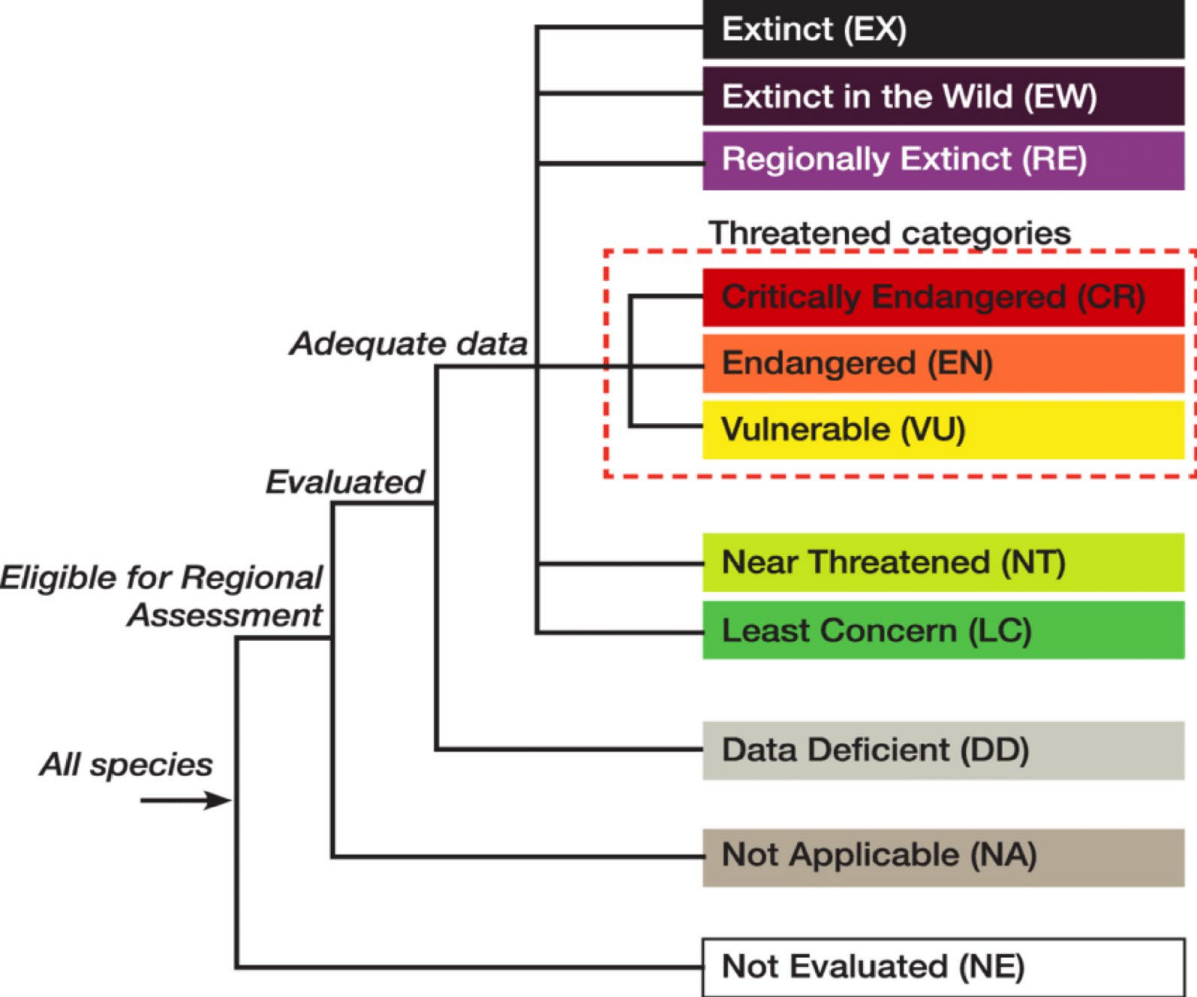
Categories of the IUCN at a regional level [70]

The field trips documented all the threats that affected the target species. Depending on the IUCN Threats Classification Scheme ver. 3.2 (https://www.iucnredlist.org/resources/classificationschemes) and [71], the threat characteristics were identified. The categorization of species is based on meeting the specific quantitative threshold for at least one of five criteria Version 3.1 was utilized (Fig. 2) [43].

### Soil analysis

Three composite soil samples were gathered randomly from the active root zones of various plants as profiles at a depth of 0–50 cm. These samples were then placed in plastic bags in the laboratory, and spread out on paper sheets to dry. After air-drying, they were sieved through a 2 mm sieve to remove gravel and debris, and subsequently stored in plastic bags for analysis. The soil texture was determined using the Bouyqucous hydrometer method to calculate the percentages of clay, silt, and sand [72]. Soil extracts preparation has been carried out by mixing soil and distilled water at a ratio of 1:5 (weight/volume) to measure Electrical Conductivity (EC), pH, carbonates, bicarbonates, chloride, sulphates, nitrates, nitrites, phosphorus, calcium, magnesium, sodium, and potassium. The glass electrode pH-meter (Jenway 3020, Cole-Parmer, Staffordshire, UK) was used for estimating the soil reaction (pH). EC (dSm^−1^) and total dissolved salts (TDS) (ppm) were assessed using a direct indicating conductivity bridge and conductivity meter. The total organic matter was detected by loss on ignition at 550^○^C [73], and estimated calcium carbonate using Bernard’s calcimeter [74]. Carbonates and bicarbonates were assessed through titration with 0.01 N HCl using phenolphthalein and methyl orange as indicators, respectively [72]. Sulphates were determined using the gravimetric method involving the ignition of residue [75]. Chloride levels were determined by direct titration against silver nitrate with 5% potassium chromate as the indicator [76–78]. Calcium and magnesium levels were determined through titration, while sodium and potassium were measured using a flame photometer. Soil extracts for available nutrient analysis were prepared with 2.5% v/v glacial acetic acid. Total nitrogen content was assessed using a Micro-Kjeldahl apparatus. Phosphorus and nitrogen levels were determined using the molybdenum blue and Indo-phenol blue methods, respectively, with a Spectrophotometer. Iron, zinc, manganese, and copper concentrations were determined using an atomic absorption method. Potassium content was assessed using a flame photometer. These processes are detailed in references [72, 79, 80].

### Prediction models

#### Environmental predictors and multicollinearity

The autocorrelation issues have been treated by eliminating redundant presences in each 1 × 1 km grid on the scale of the bioclimatic variables used. In addition, data in ArcGIS 10.6 was examined for spatial autocorrelation through average nearest neighbor analyses to eliminate spatially correlated data points [81, 82]. After this selection, the remaining occurrence points were used to generate SDMs.

Forty-five environmental factors were employed as predictors. The predictors comprised 8 precipitation measurements and 11 temperature metrics at a 30 arc-second resolution (approximately 1 km) sourced from the WorldClim database (1950–2000); version 2.1; [83]. These variables were previously utilized to estimate the distribution of plant species, encompassing seasonality, extreme climatic conditions, and regional variations in annual means (e.g [84, 85]). The data for average, minimum, and maximum values of solar radiation, precipitation, and wind speed for each month were carefully examined. The elevation data was obtained from the USGS National Elevation Dataset version 3.0, which was updated in January 2022. The research area’s topography features were modelled using the data (https://www.usgs.gov). From elevation data, slope and aspect were extracted using ArcGIS 10.8. The overall environmental variables are summarized in Table 1. Moreover, eleven soil variable were obtained from Soilgrid database (https://soilgrids.org/), while aridity index and climatic moisture content were obtained from ENVIREM database (https://envirem.github.io/; accessed 10 January 2024 [86, 87].

**Table 1:** Environmental variables used for modeling the potential distribution of the studied taxa

Variable	Code	Source	Units
Climatic/Bioclimatic variables			
Annual mean temperature	Bio1	WorldClim	°C
Mean diurnal range (max. Temp- min. temp)	Bio	WorldClim	°C
Isothermality (Bio2/Bio7) × 100	Bio3	WorldClim	°C
Temperature seasonality (SD × 100)	Bio4	WorldClim	°C
Max temperature of warmest month	Bio5	WorldClim	°C
Min temperature of coldest month	Bio6	WorldClim	°C
Temperature annual range (Bio5-Bio6)	Bio7	WorldClim	°C
Mean temperature of wettest quarter	Bio8	WorldClim	°C
Mean temperature of driest quarter	Bio9	WorldClim	°C
Mean temperature of warmest quarter	Bio10	WorldClim	°C
Mean temperature of coldest quarter	Bio11	WorldClim	°C
Annual precipitation	Bio12	WorldClim	mm
Precipitation of wettest month	Bio13	WorldClim	mm
Precipitation of driest month	Bio14	WorldClim	mm
Precipitation seasonality	Bio15	WorldClim	mm
Precipitation of wettest quarter	Bio16	WorldClim	mm
Precipitation of driest quarter	Bio17	WorldClim	mm
Precipitation of warmest quarter	Bio18	WorldClim	mm
Precipitation of coldest quarter	Bio19	WorldClim	mm
Minimum temperature	tmin	WorldClim	°C
Maximum temperature	tmax	WorldClim	°C
Average temperature	tavg	WorldClim	°C
Precipitation	Prec	WorldClim	mm
Wind speed	Wind	WorldClim	m s^−1^
Water vapor pressure	Vapr	WorldClim	kPa
Climatic moisture content		ENVIREM	-
Solar radiation	Srad		kJ m^−2^ day^−1^
Degree of water deficit below water need	Aridity index	ENVIREM	-
Topographic variables			
Elevation	Elev (m)	WorldClim	m
Slope	SL (%)	Derived from elevation	(%)
Aspect	AS (degrees)	Derived from elevation	degree
Soil factors			
Organic carbon density		Soilgrid	g/dm^3^
Soil organic carbon stock		Soilgrid	g/m²
Bulk Density		Soilgrid	cg/cm^3^
Caly content		Soilgrid	g/kg
Coarse fragment		Soilgrid	cm^3^/dm^3^
Sand		Soilgrid	g/kg
Silt		Soilgrid	g/kg
Cation exchange capacity		Soilgrid	mmol(c)/kg
Nitrogen		Soilgrid	cg/kg
Soil organic carbon		Soilgrid	g/kg
pH water		Soilgrid	pH × 10
Vol. water content at −10kpa	Water10	Soilgrid	m³/m³
Vol. water content at −33kpa	Water33	Soilgrid	m³/m³
Vol. water content at −1500kpa	Water1500	Soilgrid	m³/m³

Soil Grids (version 2.0, compiled in October 2021) provided data on soil characteristics including pH, bulk density of the fine earth fraction, volumetric fraction of coarse fragments (> 2 mm), cation exchange capacity, proportion of sand particles (> 0.05 mm) and clay particles (< 0.002 mm) in the fine earth fraction, total nitrogen, organic carbon content, and the percentage of silt particles (≤ 0.05 mm and ≥ 0.002 mm) in the fine earth fraction. The global soil data platform is available for access at https://soilgrids.org. Collaborating with multiple partners, the International Soil Reference and Information Centre (ISRIC) established this comprehensive soil information system, as per the report by Batjes et al. [88]. The Soil Grids and USGS elevation data underwent resampling to a 1 km x 1 km cell size projection, pixel location, spatial resolution of 30 arc seconds, and the extent of the bioclimatic variables utilizing ArcGIS 10.2 software**]** before analysis [89]. This step was taken to ensure spatial consistency among the 45 predictors. Additionally, we employed the capabilities of ArcGIS to trim the soil, elevation, and bioclimatic layers based on the study area shapefile.

To reduce overfitting, highly correlated variables were identified and eliminated from the SDM models by using the variance inflation factor (VIF) to evaluate the strength of each predictor in relation to the other predictors [90]. Using the vifcor and vifstep functions of the package “usdm” in R 4.2.3 [91], we carried out VIF analysis in accordance with the suggestions made by Guisan et al. [92]. With the help of these functions, we may eliminate variables that have VIF values higher than five and a 0.75 correlation criterion. The relative importance of the predictor variables was determined using the getVarImp function of the “SDM” package in R 4.3.1 [90].

We chose the IPSL-CM6A-LR global general circulation model (GCM) to examine the impact of climate change scenarios. We utilized the average of two GCM outputs from the near future (2041–2060) and the far future (2061–2080) for two different socioeconomic scenario pathways (low pathway: SSP126 and high pathway: 585), and found that this approach performed better than using results from a single model [93]. In order to project the range of the two species in response to climate change, we utilized the IPSL-CM6ALR global general circulation model (GCM). This model was used because it captured the observed present climate well and depicted the spatial patterns of global and zonal precipitation and temperature distribution quite well. The GCMs provide an accurate simulation of global warming and the multi-decadal variation in temperature and precipitation, predicting a higher increase in the mean annual temperature than other models over the same time interval [94, 95]. In previous studies, a set of four climate modeling pathways (2.6, 4.5, 6.0, and 8.5) known as Representative Concentration Pathways (RCPs) were used in long-term and near-term modeling experiments [96]. These four RCPs have been designed to describe the various amounts of greenhouse gas emissions as well as potential future radiative forcings [97]. Despite the fact that RCPs disregard socioeconomic aspects, other paths that do incorporate socioeconomic factors have been developed and are known as Shared Socioeconomic paths [97].

#### Ensemble model Building

##### R model

Four modelling algorithms—including the Generalised Linear Model (GLM), Random Forest (RF), Boosted Regression Trees (BRT) and Support Vector Machine (SVM) —were used in this study due to their high stability and the transferability of their predictions compared to other models. They are available at the’sdm’ package in R 4.3.1 [90]. The modelling algorithms included the generalized linear model (GLM: [98]) as parametric technique, the Boosting Regression Trees (BRT: [90, 99]) and the random forests (RF: [100, 101]) as non-parametric machine-learning techniques. Support Vector Machines (SVMs) are a machine learning method frequently used to build binary classifiers, also in ecological modelling [102–104]. The selected model approaches are characterized by high stability and transferability compared to other models [105–107]. Furthermore, GLM and RF behave best on both cross validation and external validation [108]. 30% of the data was used for testing, and 70% was used for training [106]. With the help of the True Skill Statistic (TSS), we weighed the ensemble models. As advised by Liuet al. [109], we applied the Maximum Training Sensitivity Plus Specificity (MTSS) criterion. We measured the TSS and the area under the receiver-operating characteristic curve (AUC) to evaluate the accuracy of the model [92]. We created binary maps (presence/absence) based on the MTSS threshold from continuous maps of the suitability of the current and future habitats to illustrate changes in the habitat.

Thus, to generate the mean ensemble of the two GCM outcomes for the distant future (2061–2080), two conjoint socioeconomic scenario pathways (high scenario: SSP585 and low scenario: SSP126(were combined. The lines that follow outline a great technique that provides better outcomes than those obtained from a single model [93, 110]. Three classes of suitability were created from the output maps of suitability under the current climate: low (< 0.3), moderate (0.3–0.5), and high (> 0.5). Furthermore, we created binary maps from continuous maps (with suitability 0.4 to 6.0) for the present and future (0: absence/1: presence) in order to visualize the changes in habitat (loss, gain, and stable areas). We then multiplied the future binary maps by 2 to create grid cells with values of (0/2), as indicated in [110].

### Extinction risk under climate and dispersal scenarios

Geometric uncertainty and issues with grid orientation or the avoidance of probable origin errors are ideal situations for applying the area of occupancy (AOO) in extinction risk assessments [111]. Full and limited dispersal scenarios should be recruited in tandem with dispersal hypotheses because they are essential components of conservation planning [106]. Both limited and complete dispersal assumptions were used to compute the AOOs. Even though the pixels were not optimal for the anticipated current range, they were kept as part of the future distribution because, under full dispersal, no restriction on the species’ dispersal abilities was assumed [110]. In line with IUCN Red List Criterion A3 (c), the reduction in the projected AOOs was utilized to evaluate the risk of extinction for species. This was determined as follows: if the loss is less than 15%, the species is considered to be of least concern (LC) [110, 112].

## Results

### Floristic analysis and IUCN red list assessment

Three taxa belonging to 3 genera and 3 families were studied (Table 2). The three taxa are home to Saint Catherine Prtotecorate (SKP). Based on the current research, the three species were classified as endangered (Table 3). Here is a comprehensive assessment of these classifications:

**Table 2:** IUCN evaluation properities used for assessing the studied endemic taxa

Scientific name	Life form	Flowering time	Pop. Size in the field	Expected Total Pop. Size (ind.)	Mat. Individuals in large subpop.	Pop. trend	System	Habitat	Soil	Uses	Associated Species
**Lamiaceae**											
*Origanum syriacum* subsp. *sinaicum*	CH	March- May, Sept.-Octo.	1500–2000	2000–3000	10–60	Decreasing	terrestrial	mountainous areas	rocky sandy soil	Md, Gr, Et, HF	*Achillea fragrantissima*,* Tanacetum sinaicum * and *Phlomis aurea*
**Plantaginaceae**											
*Anarrhinum forskaohlii* subsp. *pubescens*	Hi	Mar.- May	800–1500	1000–2500	10–50	Decreasing	terrestrial	mountainous areas	gravelly and rocky	Md, Gr, Fu,	*Achillea fragrantissima*,* Tanacetum sinaicum*,* Juncus rigidus*,* Mentha longifolia*,* Nepeta septemcrenata*, ,* Phlomis aurea*
**Polygalaceae**											
*Polygala sinaica* var. *sinaica*	CH	April- Aug.	40–100	50–200	2–10	Decreasing	terrestrial	mountainous areas	crevices of rocky areas	Md, Gr	*Achillea fragrantissima*,* Tanacetum sinaicum*,* Mentha longifolia*,* Bufonia multiceps *and* Phlomis aurea*

Life forms are represented by the following codes: CH for chamaephyte, HE for hemicryptophyte, GH for geophyte-helophyte, TH for therophyte, and Ge for geophyte. The different uses are categorized as follows: Md for medicinal, Gr for grazing, Fu for fuel, and Et for aesthetic uses

**Table 3:** Assessment of the red list of the studied taxa with the previous studies

Scientific name	Previous studies				Present study	IUCN criteria	Justification		No. of locations	Sector
	I	II	III	IV			EOO (km^2^)	AOO (Km^2^)		
**Lamiaceae**										
*Origanum syriacum* subsp. *sinaicum*	EN	EN	-	-	EN	EN B1ab (i, iv, v) + 2ab (i, iv, v)	1542.2	156	2 (fragmented)	High mountain areas, Gebel serbal area
**Plantaginaceae**										
*Anarrhinum forskaohlii* subsp. *pubescens*	CR	EN	EN	EN	EN	EN B1ab (i, iii, iv, v) + 2ab (i, iii, iv, v); CR2a(i)	880.117	144	2	High mountain areas, Gebel serbal area
**Polygalaceae**										
*Polygala sinaica* var. *sinaica*	VU	-	-	EN	EN	EN B1ab (i, iv, v) + 2ab (i, iv, v)	901.618	88	2 (fragmented)	High mountain areas, Gebel serbal area

These studies are coded as: I:[30],II:[113], and III : [114]and IV: Recent individual studies. EN: endangered, VU: vulnerable, LC: least concern, RA: rare, IN: indeterminate, CR: critically endangered, EX: extinct, DD: data deficient and NE: non-evaluated. AOO: Area of occupancy and EOO: Extent of occurrence

#### *Anarrhinum forskaohlii * subsp. pubescens D.A.Sutton

*Anarrhinum forskaohlii* subsp. *pubescens* is a perennial herb that is utilized as a medicinal plant, for grazing, and as fuel. It is home to the SKP. It is found in two specific locations within the SKP (high mountain, Serbal area). The key sites for the dissemination of this species in SKP are: Wadi Gebal, AL-Gebel Al-Ahmar, Wadi Abu tweita and Wadi Al-Arbain. Its extent of occurrence (EOO) is 880.117km^2^, and its area of occupancy (AOO) is 144 km^2^ (Table 3). It is classified as endangered (EN) based on criteria EN B1ab (i, iii, v) + 2ab (i, iii, v); CR C2a(i). The anticipated overall population is estimated to be between 1000 and 2500 mature individuals. There are distinct subpopulations, each containing 10–50 mature individuals, and it is found in sandy rocky soils within high mountainous regions. Throughout the field investigation, this particular species was predominantly confined to rocky regions in mountainous areas associated with *Achillea fragrantissima*,* Tanacetum sinaicum*,* Juncus rigidus*,* Mentha longifolia*,* Nepeta septemcrenata*, * Origanum syriacum* subsp. * sinaicum*, * Phlomis aurea*,* Alkanna orientalis* and *Stachys aegyptiaca* (Table 2). It is located in sandy terrains containing 98% sand, 1% silt, and 1% clay. It thrives in soils with a slightly elevated level of soluble salts and a moderate concentration of organic material (2.6%). EC of the soil showed a slight high percentage of total soluble salts (EC = 0.84 dS m^−1^, TDS = 538 ppm). The majority of the nutrients that are accessible exhibit minimal levels (Table 4).

#### *Origanum syriacum* subsp. *sinaicum* (Boiss.) Greuter & Burdet

It is a subshrub or herb, used as medicinal plant and is grazed by animals. In addition, it may be contributed to several industries such as: Perfume and oil. Moreover, it may be used as an esthetic plant due to the bright color of its flowers. It is distributed in two locations within SKP (High Mountains Area and Serbal Mountain). The key sites for the dissemination of this species in SKP are: El-Zawatin, W. Elarbain, W. Elfaraa, W. telah, W. El-talaa, and Farsh El-Romana. The range size (EOO) is 1542.2km^2^, and the occupied area (AOO) is 156 km^2^ (Table 3). It has been categorized as endangered (EN) based on criteria EN B1ab (i, iv, v) + 2ab (i, iv, v). The estimated total population is between 2000 and 3000 mature individuals. The subpopulations are distinctly isolated. Each subpopulation has a mature population size ranging from 10 to 60 individuals. It is confined to the rocky sandy soil of mountainous regions and wadis associated with *Achillea fragrantissima*,* Tanacetum sinaicum* and * Phlomis aurea*(Table 2). The species is typically located in sandy soils containing 89% sand, 5% silt, and 6% clay. These soils are characterized by high levels of soluble salts and a moderate amount of organic matter (2.1%). EC of the soil showed a slight high percentage of total soluble salts (EC = 1.03 dS m^−1^, TDS = 659 ppm). The majority of the nutrients that are accessible exhibit minimal levels (Table 4).

#### *Polygala sinaica* botsch var. sinaica 

*Polygala sinaica* var. *sinaica* is a perennial subshrub, used as medicinal plant and is grazed by animals. It is dispersed across two different sites within SKP (High Mountains Area and Serbal Mountain. The key sites for the dissemination of this species in SKP are: El-Zawatin, W. Elarbain, W. Elfaraa, W. Telah, W. El-Talaa, and Farsh El-romana. It has an extent of occurrence (EOO) of 901.618 km^2^ and an area of occupancy (AOO) of 88 km^2^ as shown in Table 3. It meets Endangered category (EN) under **EN B1ab (i**,** iv**,** v) + 2ab (i**,** iv**,** v)**. The estimated total population ranges from 50 to 200 mature individuals. The subpopulations are distinctly isolated,

with each containing 2–10 mature individuals. It is confined to the crevices of rocky areas of mountains associated with *Achillea fragrantissima*,* Tanacetum sinaicum*,* Bufonia multiceps and Phlomis aurea* **(**Table 2**).** The species is typically located in sandy soils characterized by 96.5% sand, 5% silt, and 1.5% clay. These soils have moderate levels of soluble salts and a low organic matter content of 0.42%. EC of the soil showed a slight high percentage of total soluble salts (EC = 0.76 dS m^−1^, TDS = 410 ppm). The majority of the nutrients that are accessible exhibit minimal levels (Table 4).

**Table 4:** Soil analysis and percentages of available nutrients of the recorded endemic taxa

Sample No.	pH	EC (dS m^−1^)	Soluble salts (meq/l)										SAR		TDS (ppm)		Gravel (%)	Particle Size Distribution [%]					SP	Organic matter		CaCO_3_
			Ca^++^	Mg^++^	Na^+^	K^+^	CO_3_^−−^	HCO_3_^−^		Cl^−^	SO_4_^−−^							Sand		Silt	Clay		%			
**1**	8.5	0.8	5.8	1.2	16.9	0.5	0.0	4.4		2.3	17.7		9.0		538		3.2	98		1.0	1.0		23	2.6		5.4
**2**	7.8	1.0	4.0	1.0	3.4	0.6	0.0	3.0		3.6	2.3		2.1		659		0.0	89		5.0	6.0		29	2.1		2.1
**3**	6.8	0.8	4.8	1.4	1.7	0.5	0.0	1.2		2.9	4.2		0.9		486		0.0	96.5		2.0	1.5		27	0.4		4.0

**Available Levels of Nutrients (p.p.m)**																										
**No.**	Species								N			P		K		Fe			Zn			Mn			Cu	
**1**	*Anarrhinum forskaohlii* subsp. *pubescens*								2.4			21		118		0.65			0.34			0.04			0.17	
**2**	*Origanum syriacum* subsp. *sinaicum*								2.6			19		121		1.10			0.28			0.09			0.13	
**3**	*Polygala sinaica* var. *sinaica*								1.3			20		112		1.10			0.17			0.16			0.31	

EC: Electric conductivity, SP: Saturation percentage and SAR: Sodium absorption value

### Modeling evaluation

#### Model performance and evolution

The RF and BRT algorithms performed better than the other algorithms. In the studied species, the ensemble models had the best overall performance with both mean TSS (above 0.86) and AUC (above 0.96) values (Table 5). Our models revealed high performance of prediction with average values of AUC (0.97 ± 0.006) for *A. forskaohlii* subsp. *pubescens*, (0.98 ± 0.007) for *O. syriacum* subsp. *sinaicum*, and (0.97 ± 0.009) for *P. sinaica* var. *sinaica* and high mean score of TSS of *A. forskaohlii* subsp. *pubescens* (0.89 ± 0.02), and *O. syriacum* subsp. *sinaicum* (0.92 ± 0.01), and *P. sinaica* var. *sinaica* (0.90 ± 0.01) and other evaluation parameters showed high values (Table 5).

**Table 5:** Performance of the model algorithms for the three studied taxa

Methods	*Anarrhinum forskaohlii* subsp. *pubescens*						*Origanum syriacum* subsp. *sinaicum*						
	BRT	GLM	RF	SVM		Average	BRT		GLM	RF		SVM	Average
**threshold**	0.28 ± 0 0.1	0.38 ± 0.09	0.26 ± 0.10	0.34 ± 0.1		0.31 ± 0.1	0.21 ± 0.04		0.28 ± 0.10	0.42 ± 0.20		0.26 ± 0.1	0.29 ± 0.10
**sensitivity**	0.93 ± 0.02	0.95 ± 0.02	0.95 ± 0.01	0.93 ± 0.02		0.94 ± 0.02	0.98 ± 0.01		0.98 ± 0.005	0.96 ± 0.01		0.97 ± 0.005	0.97 ± 0.006
**specificity**	0.95 ± 0.01	0.94 ± 0.02	0.94 ± 0.02	0.94 ± 0.01		0.94 ± 0.01	0.93 ± 0.02		0.93 ± 0.005	0.96 ± 0.01		0.94 ± 0.01	0.94 ± 0.01
**TSS**	0.89 ± 0.01	0.89 ± 0.03	0.89 ± 0.01	0.88 ± 0.02		0.89 ± 0.02	0.92 ± 0.005		0.91 ± 0.00	0.94 ± 0.01		0.92 ± 0.01	0.92 ± 0.01
**Kappa**	0.86 ± 0.02	0.83 ± 0.04	0.84 ± 0.05	0.84 ± 0.03		0.84 ± 0.03	0.88 ± 0.02		0.88 ± 0.00	0.92 ± 0.02		0.89 ± 0.01	0.89 ± 0.02
**Overall accuracy**	0.95 ± 0.005	0.94 ± 0.01	0.94 ± 0.02	0.94 ± 0.01		0.94 ± 0.01	0.94 ± 0.01		0.95 ± 0.01	0.96 ± 0.01		0.95 ± 0.005	0.95 ± 0.01
**AUC**	0.97 ± 0.005	0.97 ± 0.005	0.98 ± 0.005	0.97 ± 0.005		0.97 ± 0.006	0.99 ± 0.005		0.97 ± 0.005	0.99 ± 0.01		0.98 ± 0.005	0.98 ± 0.007

Methods	*Polygala sinaica* var. *sinaica*												
	BRT		GLM		RF			SVM			Average		
**threshold**	0.15 ± 0.12		0.18 ± 0.08		0.26 ± 0.19			0.06 ± 0.02			0.16 ± 0.1		
**sensitivity**	0.96 ± 0.02		0.95 ± 0.01		0.94 ± 0.04			0.96 ± 0.005			0.95 ± 0.02		
**specificity**	0.95 ± 0.02		0.94 ± 0.01		0.96 ± 0.01			0.94 ± 0.01			0.95 ± 0.01		
**TSS**	0.91 ± 0.005		0.90 ± 0.01		0.90 ± 0.02			0.90 ± 0.01			0.90 ± 0.01		
**Kappa**	0.83 ± 0.04		0.80 ± 0.02		0.84 ± 0.04			0.79 ± 0.02			0.81 ± 0.03		
**Overall accuracy**	0.95 ± 0.01		0.94 ± 0.005		0.95 ± 0.01			0.94 ± 0.01			0.94 ± 0.01		
**AUC**	0.98 ± 0.005		0.96 ± 0.005		0.98 ± 0.005			0.97 ± 0.005			0.97 ± 0.009		

**Fig. 3:**
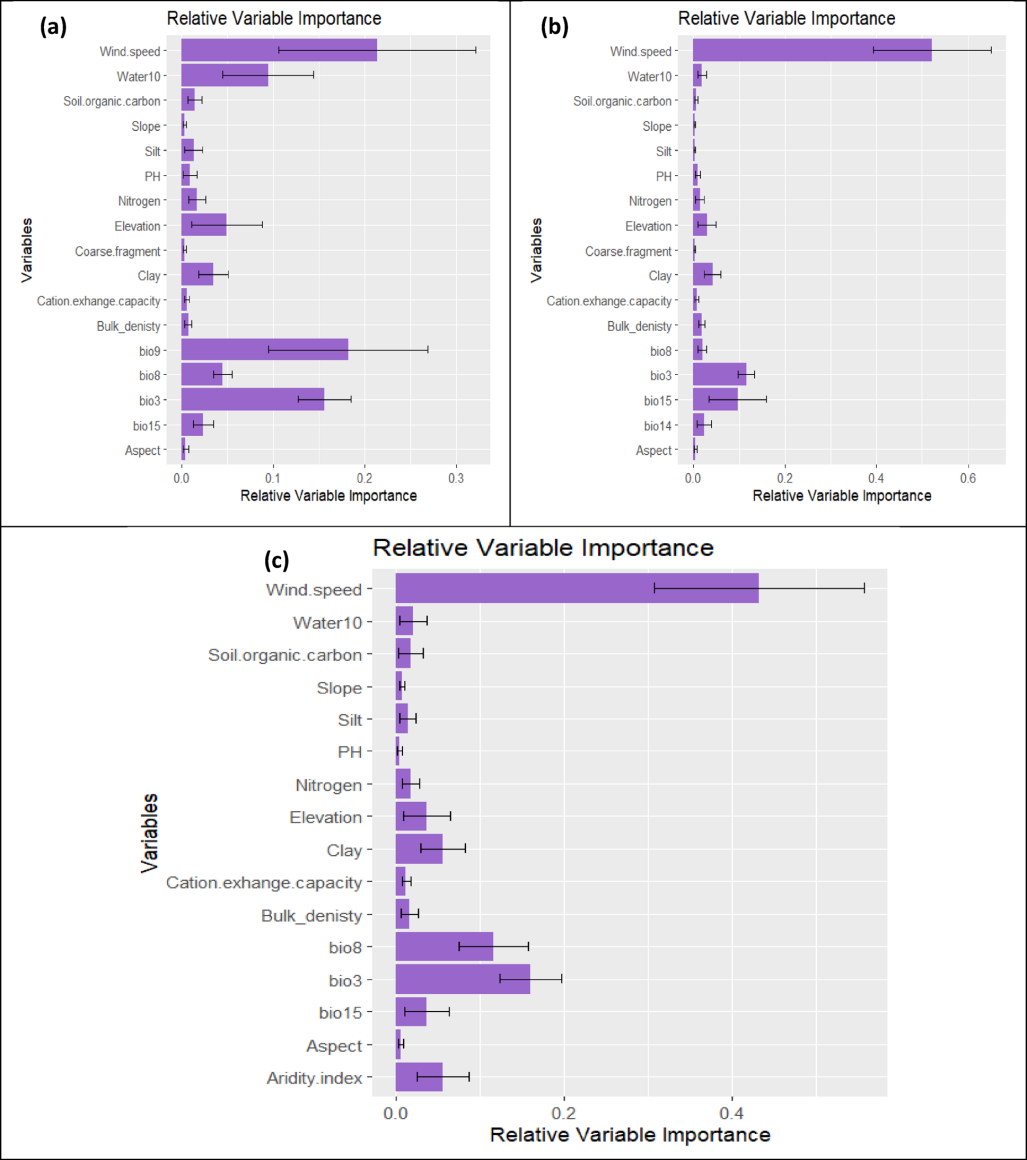
Relative variable importance of the selected environmental variables used in the ensemble models for predicting the potential distribution of (**a**) *A. forskaohlii* subsp. *pubescens*, (**b**) *O. syriacum* subsp. *sinaicum*, and (**c**) *P. sinaica* var. *sinaica* under current climate conditions

Analysis of multicollinearity among the [45] predictors **(**Table 1**)** revealed that 14 variables were uncorrelated and have VIFs lower than 5 for the three taxa (Table 6). These variables were utilized in the ensemble modeling process. According to Pearson’s correlation coefficient, the relative importance of the predictor variables contributing to the ensemble model showed Wind, Bio9, Bio3, water 10 and elevation were the most contributing variables that control the distribution of *A. forskaohlii* subsp. *pubescens* with contributing variable equals 21.4, 18.2, 15.6, 9.5 and 4.9%, respectively, the most contributing variables that control *O. syriacum* subsp. *sinaicum* were wind (47.9%), Bio3 (13.2%), Bio15 (11.9%), clay (4.4%), and elevation (3.2%). While wind, Bio3, Bio8, clay, aridity index and elevation were the most contributing variables that control the distribution of *P. sinaica* var. *sinaica* with contributing variable equals 43.3, 16, 11.6, 5.5, 5.5 and 3.6%, respectively **(**Fig. 3**).**

**Fig. 4:**
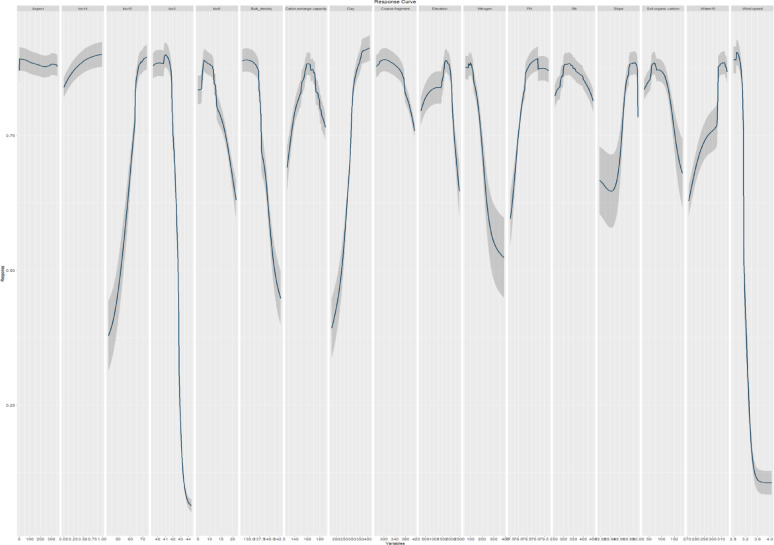
The response curves of the predictor variables employed in the distribution modeling of *A. forskaohlii* subsp. *pubescens*

**Fig. 5:**
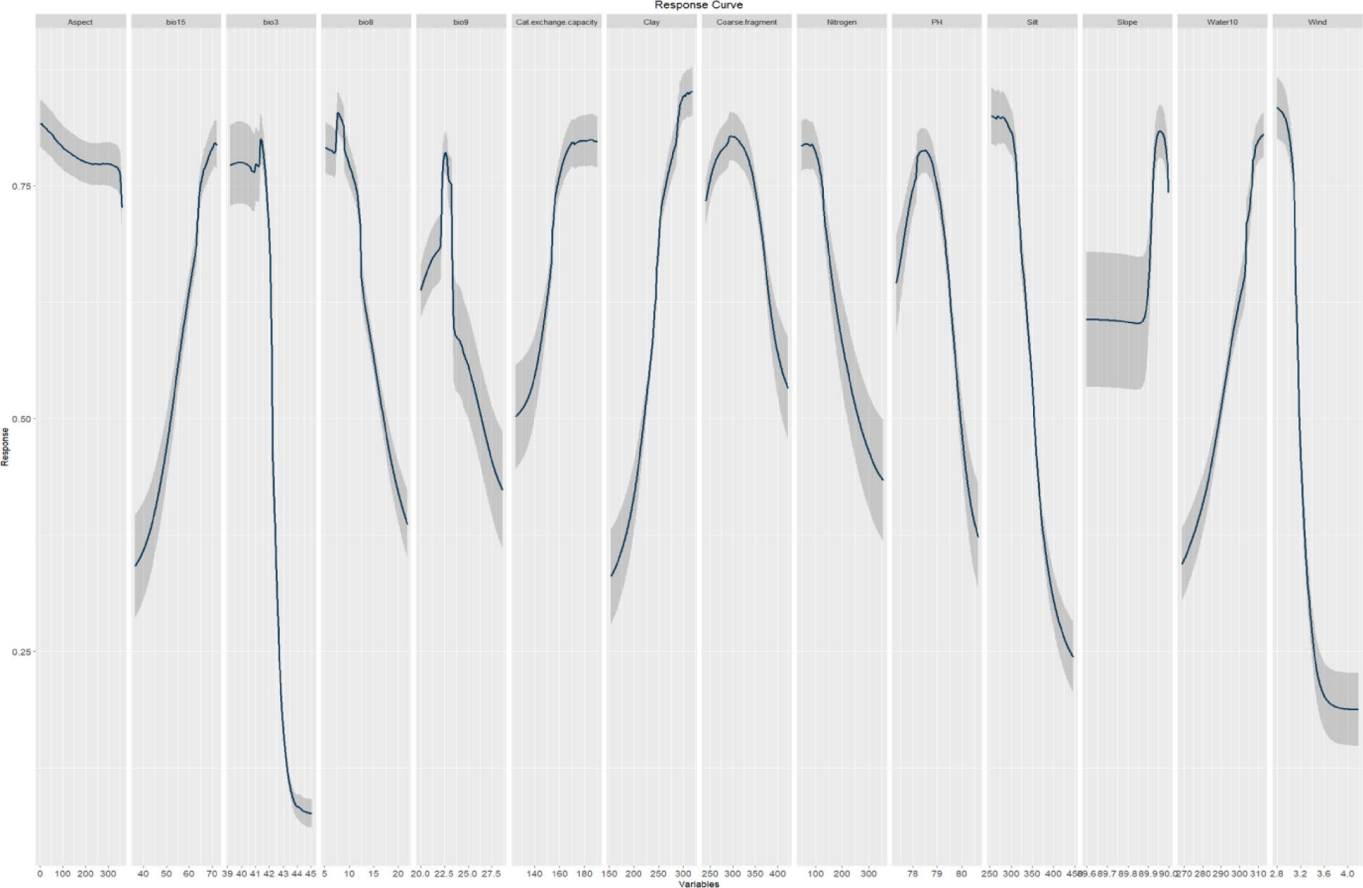
The response curves of the predictor variables employed in the distribution modeling of *O. syriacum* subsp. *sinaicum*.

The response curves revealed the relationship between predictive variables and the logistic prediction of habitat suitability **(**Figs. 4, 5**)**. The response curves of *A. forskaohlii* subsp. *pubescens* indicate that as clay Bio15, slope and water10 rise, the likelihood of presence also increases (Fig. 4**).** Conversely, the likelihood of presence decreases with higher Bio3, Bio8, Bio9, coarse fragment, Nitrogen, silt, soil organic carbon and wind. On the othe hand, likelihood of presence also increases with increasing pH and cation exchange capacity to specific extent, then it decreases again (Fig. 4**).**

**Fig. 6:**
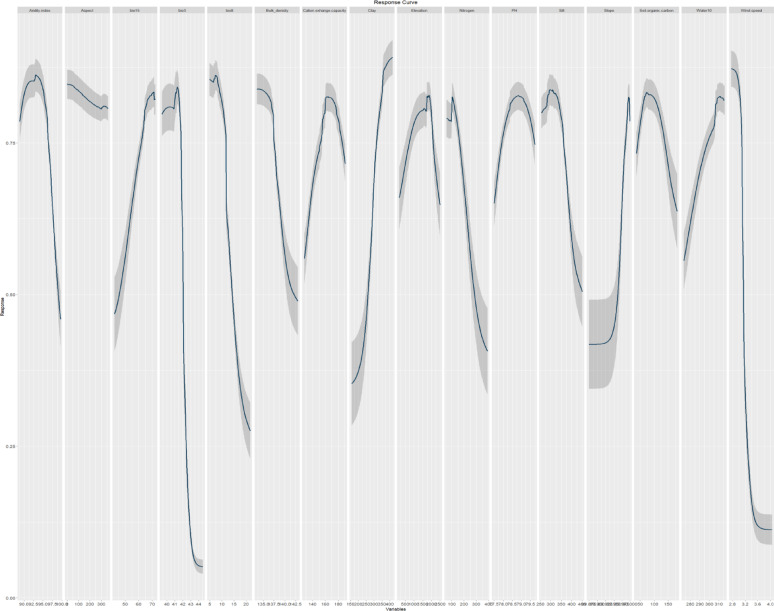
The response curves of the predictor variables employed in the distribution modeling of *P. sinaica *var.* sinaica*

Regarding *Origanum syriacum* subsp. *sinaicum*, response curves demonstrated that the increase in Bio15, clay, pH, and water10 was associated with a higher probability of presence (Fig. 5). Conversely, the probability of presence decreased as Bio3, Bio8, Nitrogen, coarse fragment, soil organic carbon, and wind increased. Moreover, likelihood of presence also increases with increasing cation exchange capacity, elevation, silt and slope to specific extent, and then it decreases again. On the other hand, the response curves of *P. sinaica* var. *sinaica* indicated a higher likelihood of presence with the increase in clay, bio15, water10 and slope (Fig. 6). In contrast, as the aridity index, Bio3, Bio8, bulk density, Nitrogen, silt and wind rise, the likelihood of presence falls. In addition, the likelihood of presence also increases with increasing cation exchange capacity, elevation, pH and soil organic carbon to specific extent, and then it decreases again.

**Table 6:** Summary of the chosen environmental predictor variables that explain the potential distribution of three studied taxa in SKP with their VIF values

*A. forskaohlii* subsp. *pubescens*					*O. syriacum* subsp. *sinaicum*				
Code	Variable			VIF	Code	Variable			VIF
Silt	Silt			3.6	**Aspect**	AS (degrees)			2.1
SL (%)	Slope			1.2	**Bio3**	Isothermality (Bio2/Bio7) × 100 (°C)			1.8
Soil organic carbon	Soil organic carbon (g/kg)			1.9	**Bio8**	Mean temperature of wettest quarter (°C)			3.7
wind speed	Wind (m s^−1^)			2.4	**Bio15**	Precipitation seasonality (mm)			3.3
Aspect	AS (degrees)			2.1	**Bulk denisty**	Bulk density (cg/cm3)			2.0
Elev (m)	Elevation			3.6	**Cation exchange**	Cation exchange capacity (mmol(c)/kg)			2.4
Bio3	Isothermality (Bio2/Bio7) × 100 (°C)			1.5	**Clay**	Clay (g/kg)			3.2
Bio8	Mean temperature of wettest quarter (°C)			2.5	**Coarse fragment**	Coarse fragment (cm^3^/dm^3^)			2.1
Bio9	Mean temperature of driest quarter (°C)			4.0	**Nitrogen**	Nitrogen (cg/kg)			3.7
Bio15	Precipitation seasonality			3.8	**pH**	pH water (pH × 10)			2.1
Clay	Clay (g/kg)			2.6	**Silt**	Silt			2.0
Coarse fragment	Coarse fragment (cm^3^/dm^3^)			4.1	**SL (%)**	Slope			3.2
Cation exchange	Cation exchange capacity (mmol(c)/kg)			2.7	**Soil organic carbon**	Soil organic carbon (g/kg)			2.4
Bulk denisty	Bulk density (cg/cm3)			2.1	**Water10**	Vol. water content at −10kpa (m³/m³)			3.2
Water10	Vol. water content at −10kpa (m³/m³)			3.3	**wind speed**	Wind (m s^−1^)			2.6
Nitrogen	Nitrogen (cg/kg)			2.4	**Elev (m)**	Elevation			3.8
pH	pH water (pH × 10)			2.0					

*P. sinaica* var. *sinaica*									
Code		Variable						VIF	
Aspect		AS (degrees)						2.0	
Degree of water deficit below water need		Aridity index						3.0	
Bio3		Isothermality (Bio2/Bio7) × 100 (°C)						1.4	
Bio8		Mean temperature of wettest quarter (°C)						3.5	
Bio15		Precipitation seasonality (mm)						2.1	
Bulk denisty		Bulk density (cg/cm3)						2.4	
Cation exchange		Cation exchange capacity (mmol(c)/kg)						2.7	
Clay		Clay (g/kg)						3.7	
Nitrogen		Nitrogen (cg/kg)						2.7	
pH		pH water (pH × 10)						2.4	
Silt		Silt						3.8	
SL (%)		Slope						1.9	
Elev (m)		Elevation						4.1	
Soil organic carbon		Soil organic carbon (g/kg)						2.5	
Water10		Vol. water content at −10kpa (m³/m³)						2.6	
wind speed		Wind (m s^−1^)						2.1	

#### Current and future predictions

**Fig. 7:**
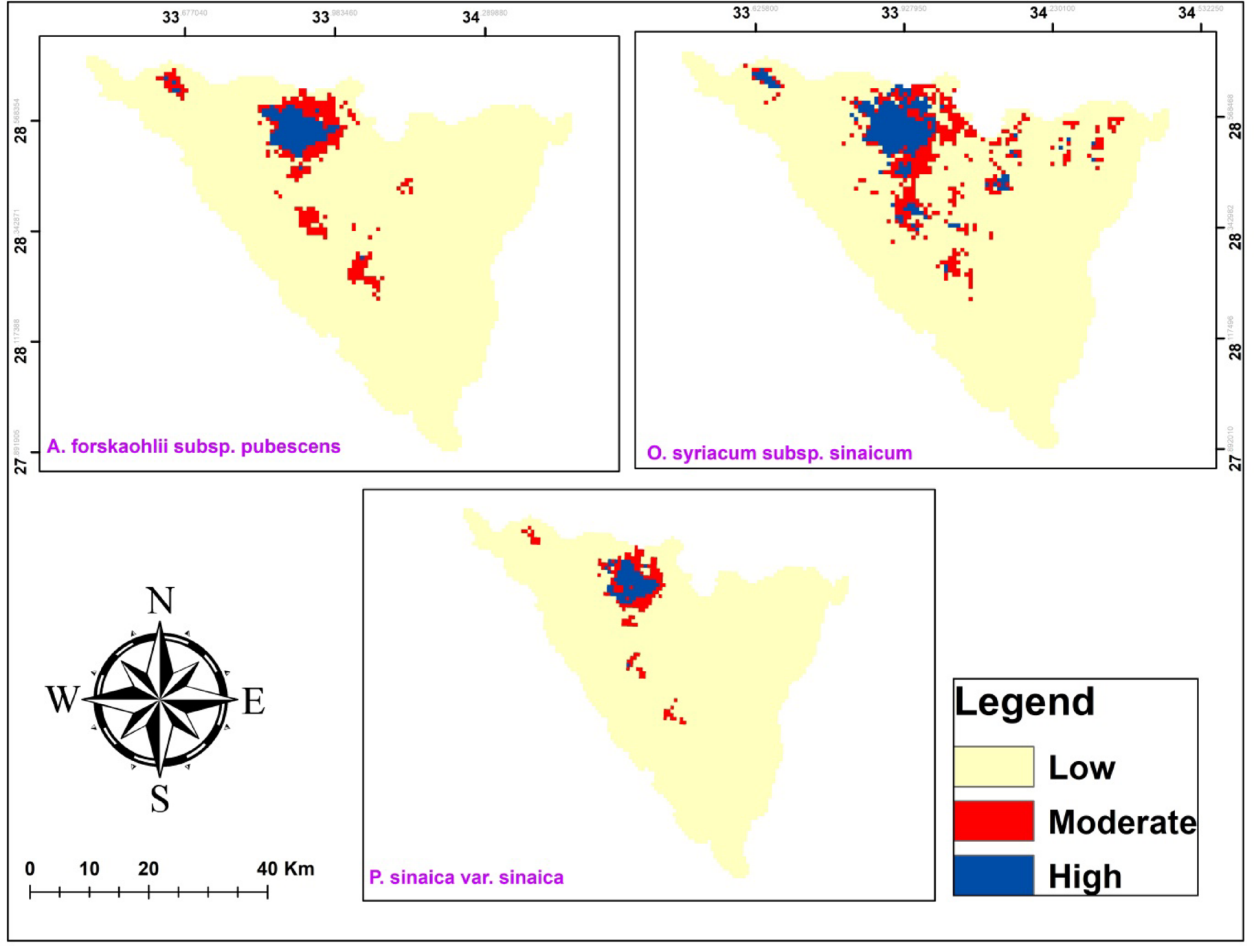
The habitat suitability map of the three studied taxa under current conditions

Ensemble habitat suitability map presented that the area of the currently suitable habitats for *A. forskaohlii* subsp. *pubescens* under MTSS threshold 0.31 was 198 km^2^. It showed high habitat suitability in southern Sinai in Wadi Gebal, AL-Gebel Al-Ahmar, Wadi Abu tweita, Gebel Musa, Gebel Catherine and Wadi Al-Arbain (Fig ). In addition, the total area of suitable habitats for *O. syriacum* under the MTSS threshold of 0.35 was calculated to be 377 km^2^. It showed high potential distribution in Saint Catherine in El-Zawatin, W. Elarbain, W. Elfaraa, W. telah, W. El-talaa, and Farsh El-Roman (Fig. 7). Moreover, the area of the presently suitable habitats for *P. sinaica* under the MTSS threshold of 0.3, as shown by the ensemble habitat suitability map, was 167 km^2^. El-Zawatin, W. Elarbain, W. Elfaraa, W. Telah, W. El-Talaa, and Farsh El-romana all displayed high species habitat suitability in Saint Catherine (Fig. 7).

**Fig. 8:**
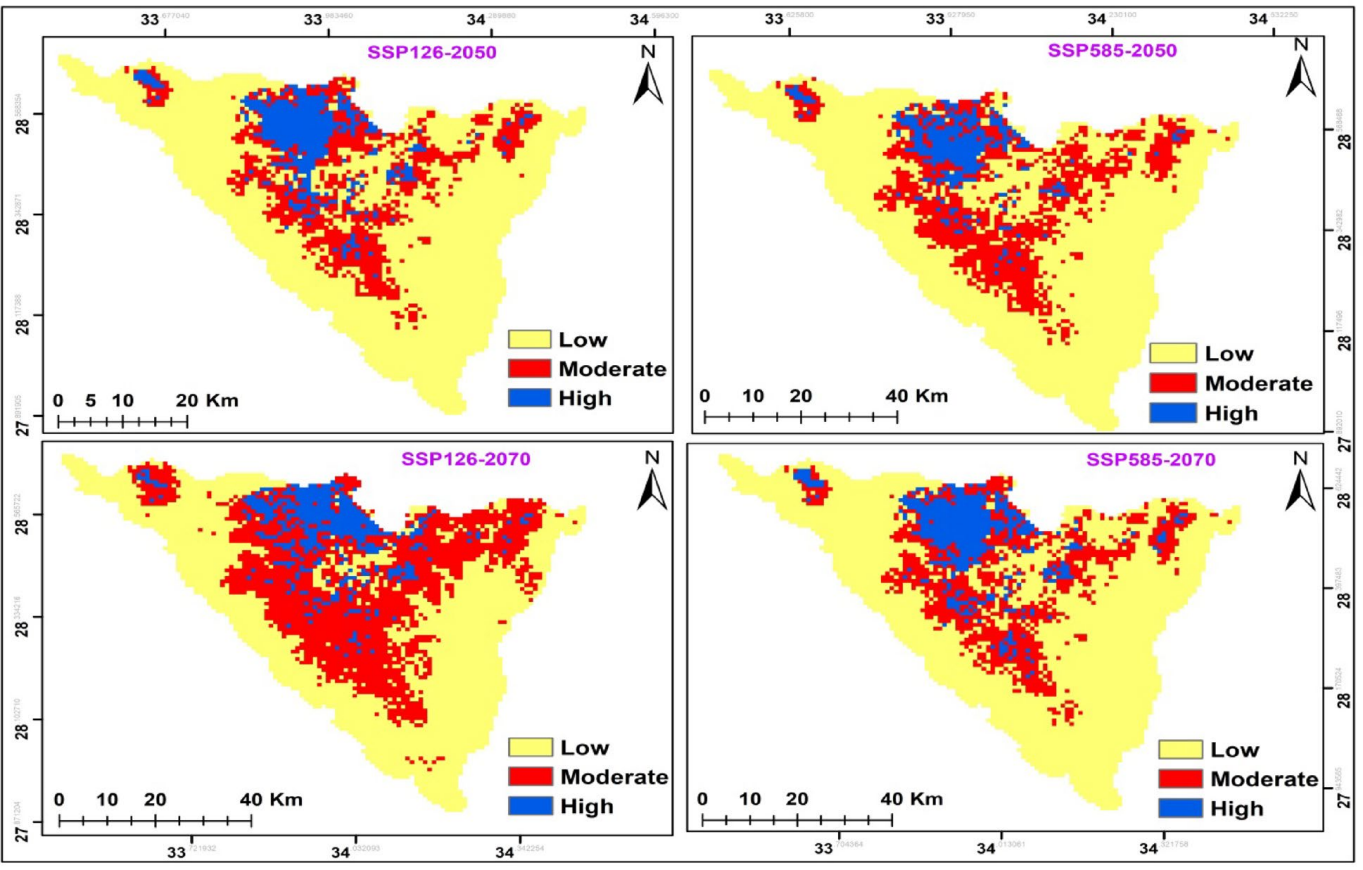
The habitat suitability map of *A. forskaohlii *subsp.* pubescens *under the two different scenarios for the two periods (2041-2060/2061-2080)

The potential modifications in habitat suitability were somewhat alike in both climate change scenarios in the two time periods (2041–2060; 2061–2080) (Fig. 8 and Table 7). In both cases, there was increase in the potential range of this species. *A. forskaohlii* subsp. *pubescens* prediction under the SSP126 scenario of the IPSL-CM6A-LR GCM model for the period 2050 and 2070 revealed a projected increase in the suitable area compared to the current distribution, with the suitable area covering 397 km^2^ and 427 km^2^ of the total study area, respectively. Otherwise, at SSP585, habitat suitability increased with climate warming for 2050 by 356 km^2^ and 357 km^2^ for 2070 period compared to the current distribution (Fig. 8 and Table 7). The loss gain areas were concentrated in of the high mountain area of the St. Katherine Protectorate in southern Sinai, especially Gebel Musa, Gebel Catherine and Serbal Mountain regions.

**Fig. 9:**
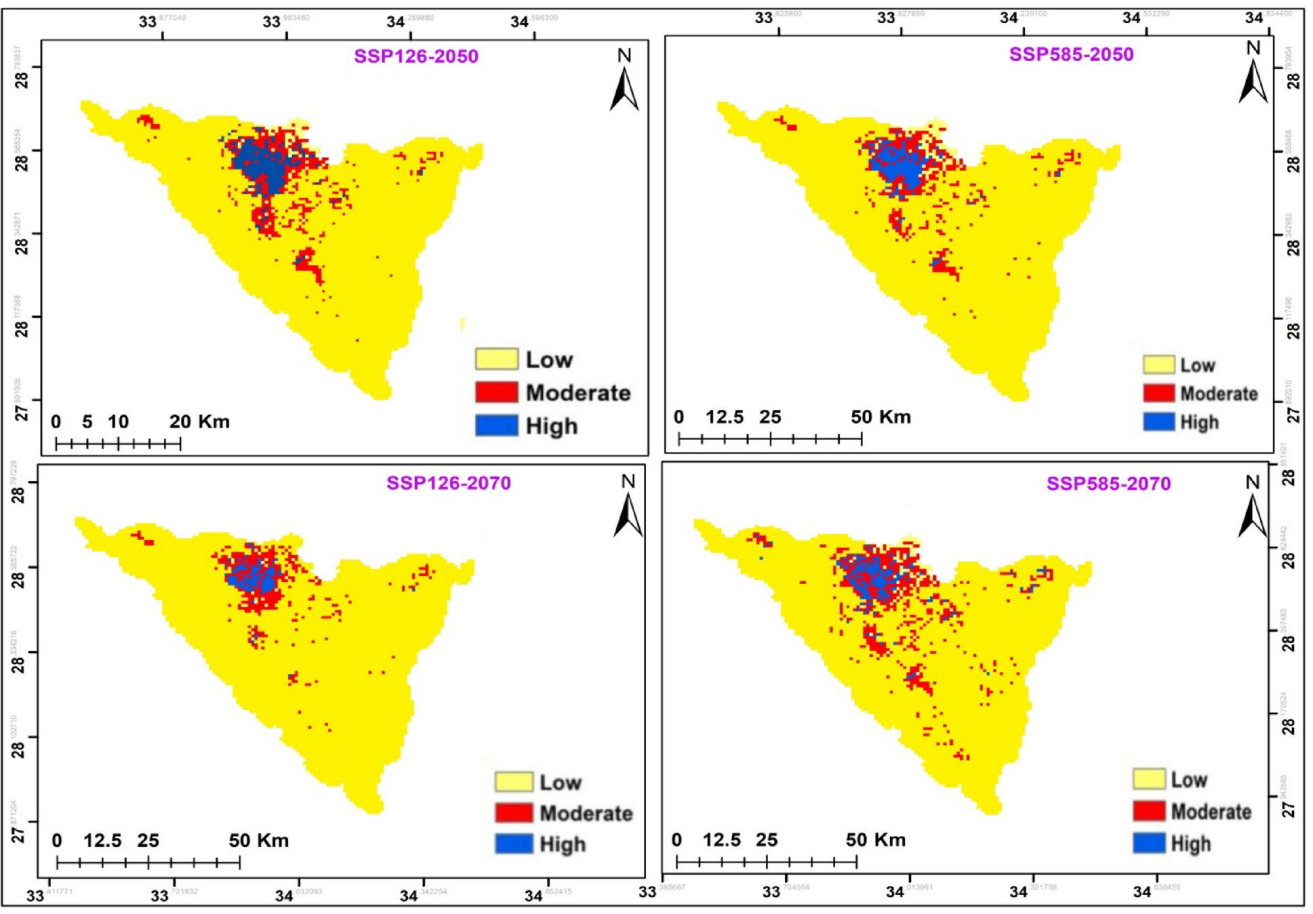
The habitat suitability map of *O. syriacum *subsp.* sinaicum *under the two different scenarios for the two periods (2041-2060/2061-2080)

The suitable area of SSP126 and SSP585 climate scenarios of the IPSL-CM6A-LR general climate model for *O. syriacum* subsp. *sinaicum* will decrease under all climate scenarios, by 2050 and 2070 (Fig. 9. and Table 7). It is predicted that the suitable habitat will decrease by 356, 326, 304, and 268 km^2^ under SSP126 (2050), SSP585 (2050), SSP126 (2070) and SSP585 (2070), respectively.

**Fig. 10:**
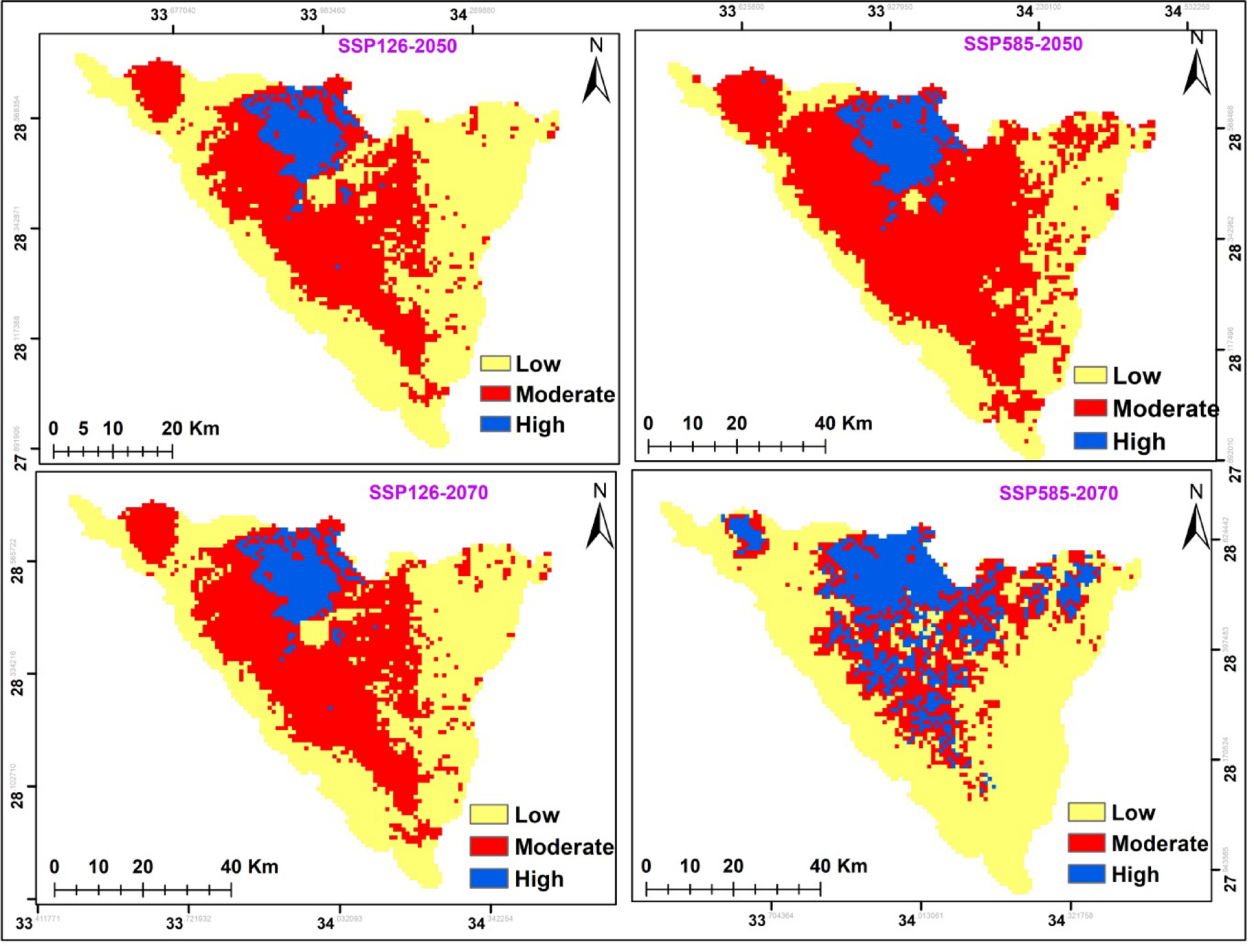
The habitat suitability map of *P. sinaica *var.* sinaica *under the two different scenarios for the two periods (2041-2060/2061-2080)

On the other hand, the suitable area of SSP126 and SSP585 climate scenarios of the IPSL-CM6A-LR general climate model for *P. sinaica* var. *sinaica* will increase under all climate scenarios, by 2050 and 2070. (Fig. 10. and Table 7). It is predicted that the suitable habitat will increase by 559, 566, 365, 546 and 617 km^2^ under SSP126 (2050), SSP585 (2050), SSP126 (2070), and SSP585 (2070), respectively.

**Table 7:** Comparison between current and future habitat suitability of the three studied taxa

*A. forskaohlii *subsp. *pubescens*										
Habitat suitability						**Habitat change**				
Suitability class	**Current**	**Future**					**Future**			
		**2041–2060**		**2061–2080**			**2041–2060**		**2061–2080**	
		SSP126	SSP585	SSP126	SSP585		SSP126	SSP585	SSP126	SSP585
Unsuitable	4847	4648	4689	4618	4688	**Loss**	12 (6%)	24 (12.1%)	13 (6.5%)	28 (14.1%)
Suitable	198 (3.9%)	397 (7.9%)	356 (7.1%)	427 (8.5%)	357 (7.1%)	**Unsuitable**	4636	4665	4605	4660
						**Stable**	186	174	185	170
						**Gain**	211 (51.5%)	182 (47.9%)	242 (55%)	187 (48.5%)

*O. syriacum* subsp. *sinaicum*										
Habitat suitability						**Habitat change**				
Suitability class	**Current**	**Future**					**Future**			
		**2041–2060**		**2061–2080**			**2041–2060**		**2061–2080**	
		SSP126	SSP585	SSP126	SSP585		SSP126	SSP585	SSP126	SSP585
Unsuitable	4668	4689	4719	4741	4777	**Loss**	148 (39.2%)	149 (39.5%)	161 (42.7%)	224 (59.4%)
Suitable	377 (7.5%)	356 (7.1%)	326 (6.5%)	304 (6.0%)	268 (5.3%)	**Unsuitable**	4541	4570	4580	4553
						**Stable**	229	228	216	153
						**Gain**	127 (25.1%)	98 (20.6%)	88 (18.9%)	115 (23.4%)

*P. sinaica* var. *sinaica*										
Habitat suitability						**Habitat change**				
Suitability class	**Current**	**Future**					**Future**			
		**2041–2060**		**2061–2080**			**2041–2060**		**2061–2080**	
		SSP126	SSP585	SSP126	SSP585		SSP126	SSP585	SSP126	SSP585
Unsuitable	4878	4486	4479	4481	4428	**Loss**	-	3 (1.8%)	1 (0.6%)	5 (3%)
Suitable	167 (3.3%)	559 (11.1%)	566 (11.2%)	564 (11.2%)	617 (12.2%)	**Unsuitable**	4486	4476	4480	4423
						**Stable**	167	164	166	162
						**Gain**	392 (70.1%)	402 (70.6%)	398 (70.4%)	455 (73.1%)

**Fig. 11:**
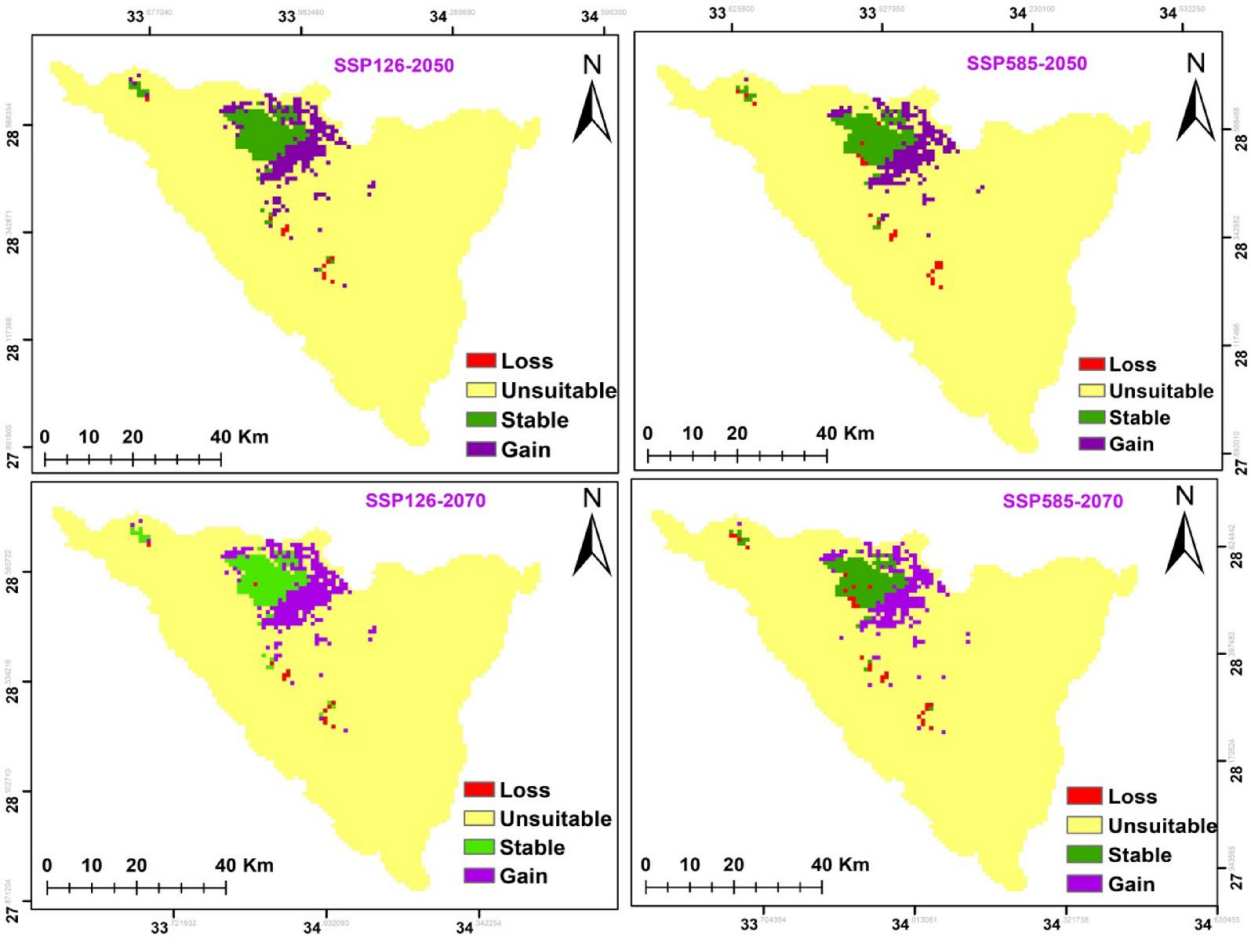
Possible habitat change under the two scenarios of climate change for *A. forskaohlii *subsp.* pubescens*

According to two different climate change scenarios, there were differences in the potential future alterations in *A. forskaohlii* subsp. *pubescens* habitat suitability. According to both forecasts, this species’ prospective range could expand under SSP126 by 211 km^2^ and SSP585 by 182 km^2^ for 2050. *A. forskaohlii* subsp. *pubescens* range revealed that 12 km^2^ of the currently suitable habitats will lose under SSP126 (the most optimistic scenario), while 24 km^2^ will be gained under SSP585. By 2070, the gained area will increase under SSP126 and SSP585 by 242 and 187 km^2^, respectively. The loss areas will slightly increase to be 13 km^2^ under SSP126 and 28 km^2^ under SSP585. The majority of the gained areas located at Shag Musa, Gebel Catherine, and Serbal Mountain regions (Fig. 11 and Table 7).

**Fig. 12:**
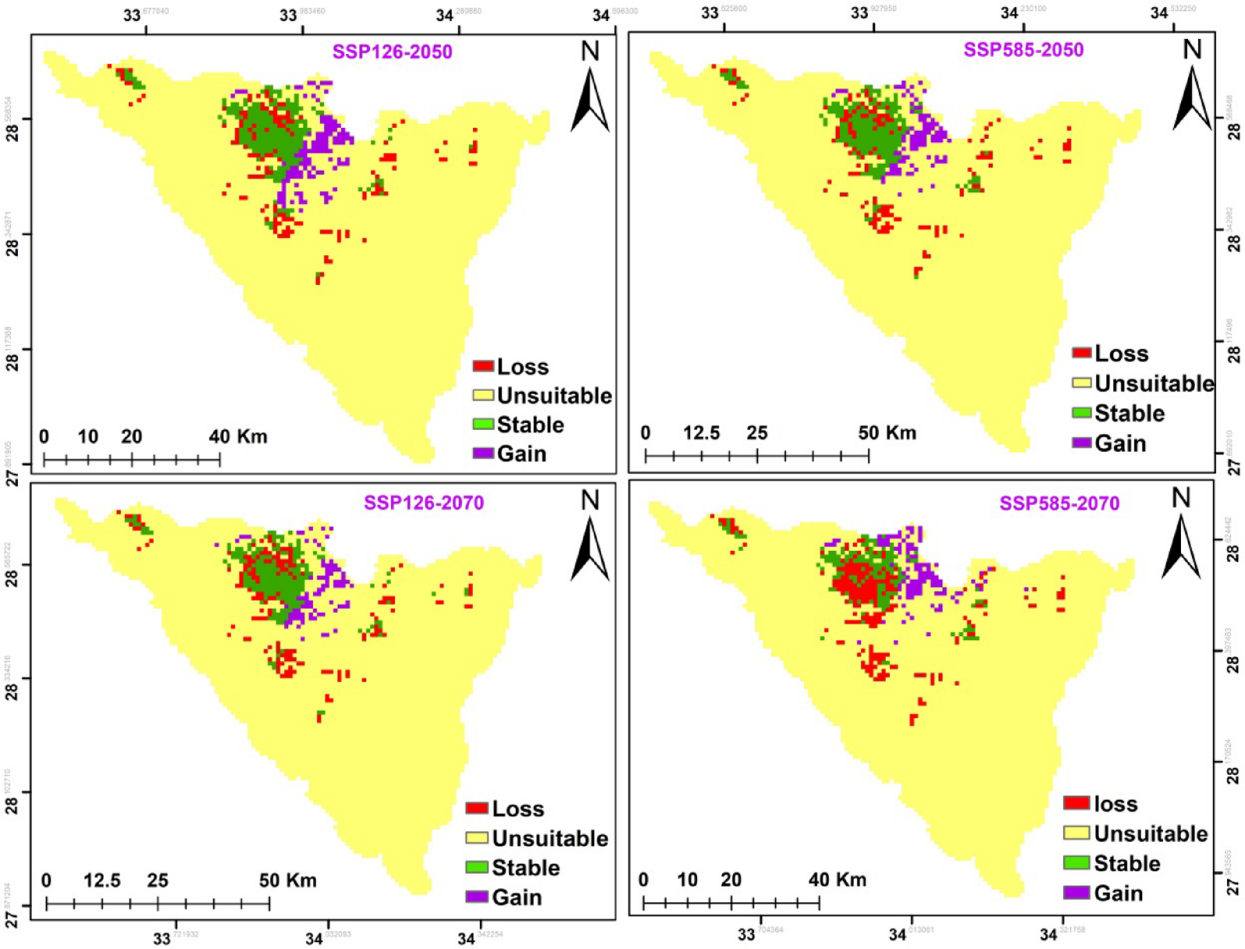
Possible habitat change under the two scenarios of climate change for *O. syriacum *subsp.* sinaicum*

Based on the results of the ensemble model, the distribution pattern of *O. syriacum* subsp. *sinaicum* was projected to change under the different climate change model with different SSPs scenarios in the near and far future as compared to the current distribution pattern. It is predicted that the species distribution range will decline by 148, 149, 161, and 224 km^2^ under SSP126 (2050), SSP585 (2050), SSP126 (2070) and SSP585 (2070), respectively. Meanwhile, it will expand by 127, 98, 88 and 115 km^2^ at SSP126 (2050), SSP585 (2050), SSP126 (2070) and SSP585 (2070), respectively. The majority of the declined areas located at El-Zawatin, W. Elarbain, Farsh El-Roman, and Serbal regions (Fig. 12 and Table 7).

**Fig. 13:**
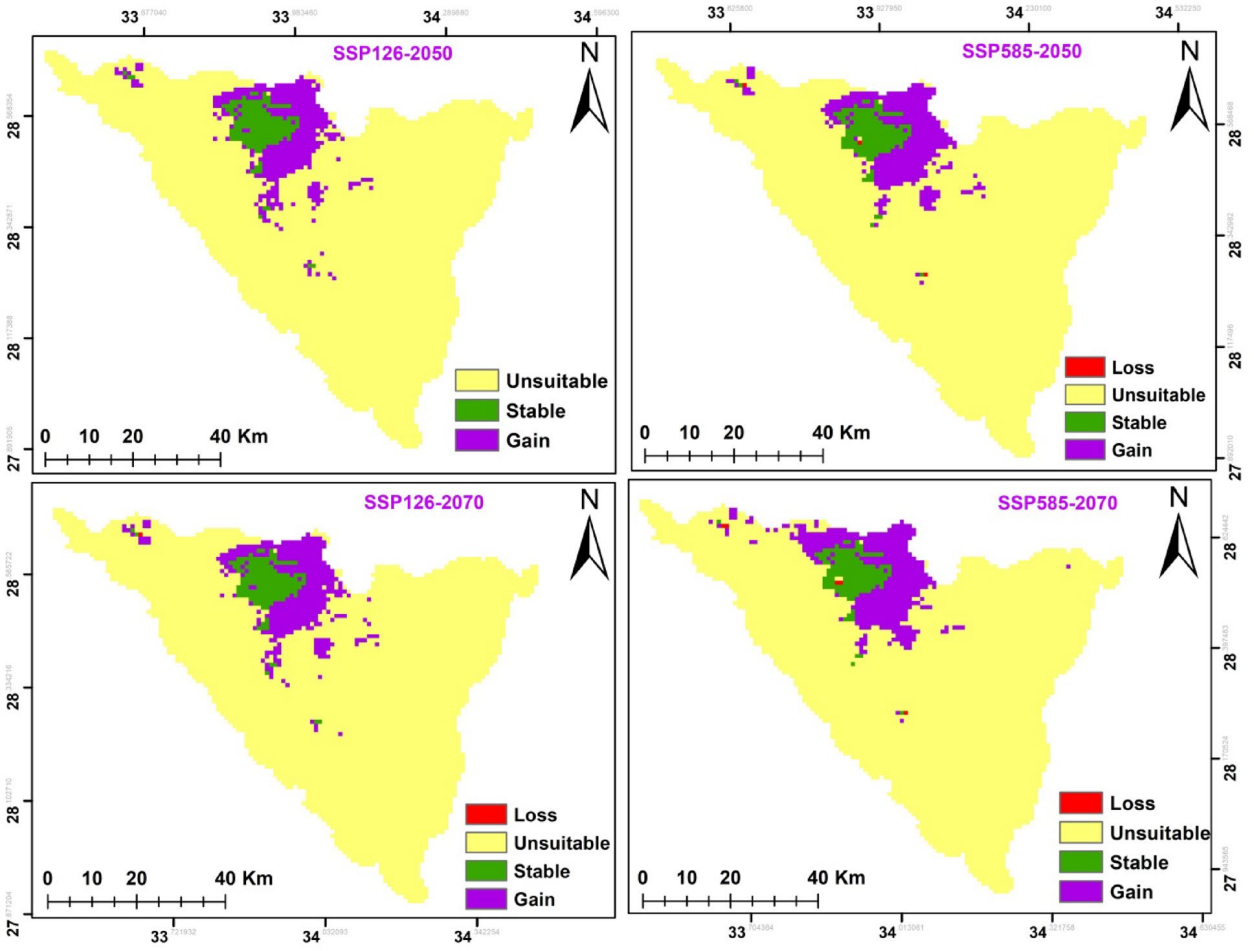
Possible habitat change under the two scenarios of climate change for *P. sinaica *var.* sinaica*

On the other hand, the distribution pattern of *P. sinaica* var. *sinaica* was projected to change under the different climate change model with different SSPs scenarios based on the results of the ensemble model. It is predicted that the species distribution range will expand by 392, 402, 398, and 455 km^2^ under SSP126 (2050), SSP585 (2050), SSP126 (2070), and SSP585 (2070), respectively. Meanwhile, the loss areas are neglectable. The majority of the expansion areas located at El-Zawatin, W. Elarbain, W. Telah, Farsh El-romana, Gebel Catherine, Musa, and serbal regions (Fig. 13 and Table 7).

### The area projected to be occupied (AOO) and potential shifts in conservation status under different climate change and dispersal scenarios

There were similarities observed in the changes in habitat suitability and the extent of loss in the Area of Occupancy (AOO) across both dispersal scenarios and under various climate change scenarios **(**Table 8**)**. The full dispersal scenario showed a slightly lower percentage of AOO loss compared to the limited dispersal scenario across all climate change scenarios (Table 2). Upon examination of the AOO loss percentage based on the IUCN Red List criterion A3(C) for both climate and dispersal scenarios (Table 2), it is expected that both *O. syriacum* subsp. *sinaicum* and *A. forskaohlii* subsp. *pubescens* will be uplisted to “Critically Endangered” due to the risk of decline under both climate change scenarios SSP126 and SSP585, covering the period from 2061 to 2080 (Table 8). whereas, *P. sinaica* var. *sinaica* will be categorized as “Critically Endangered” under SSP 126 and remain “Endangered” under SSP585.

**Table 8:** Loss percentage in the area of occupancy (AOO) of the studied species under the two climate change scenarios and the two dispersal scenarios

Species	AOO Loss %		Current IUCN status	Proposed IUCN status
	Full Dispersal	Limited Dispersal		
*Origanum syriacum* subsp. *sinaicum*			EN	
SSP126 (2061–2080)	0	0		CR
SSP585 (2061–2080)	0	0		CR
*Polygala sinaica* var. *sinaica*			EN	
SSP126 (2061–2080)	0	0		CR
SSP585 (2061–2080)	0	0		EN
*Anarrhinum forskaohlii* subsp. *pubescens*			EN	
SSP126 (2061–2080)	2.7	7.7		CR
SSP585 (2061–2080)	1.2	5		CR

Proposed conservation status according to IUCN Red List criterion (AOO). LC is the least concern status, while EN means endangered

## Discussion

Understanding the spatial distribution of biodiversity and endemism is crucial for effective conservation planning [115], particularly given the rapid transformation of landscapes [116] and the impact of climate change [117]. Endemic species are those with limited geographical ranges and specialized ecological niches [118]. When compared to certain countries in the Middle East, Egypt has a lower-than-average level of endemism, similar to other arid southern countries [119].

### IUCN Red List assessment

Using the IUCN Red List criteria, the three taxa were assessed as endangered species. *Anarrhinum forskaohlii* subsp. *pubescens* is evaluated as endangered in the present study and [66, 113, 114], but critically endangered by Hosni et al. [30]. It occupies a restricted mountainous in south Sinai. This species is distributed in two locations due to long-term drought and the destructive effect of sudden flooding [66]. There is a continuing decline in its habitat quality, with evidence of declines in the numbers of subpopulation and mature individuals. The mountainous habitat serves as a barrier between small sub-populations, leading to fragmentation of the population. Many of these sub-populations have low viability because of over-grazing, which results in the loss of reproductive organs. A severe impact had arisen due to the touristic development in its area of occurrence [32, 66, 120, 121]. In addition, Overgrazing cause destruction of the reproductive organs of *A. forskaohlii* subsp. *pubescens* and decrease the opportunity for the possibility of producing new generation [122]. *Origanum syriacum* subsp. *sinaicum* is evaluated as endangered in the present study as well as Hosni et al. [30] and [113]. *Polygala sinica* var. *sinaica* is evaluated as endangered in the present study like Omar et al. [69], but vulnerable by Hosni et al. [30]. Drought is the major threat to this species’ distribution in SKP. Overgrazing will be most harmful during droughts, and it may lead to a decrease in population size over time. Climate change may pose a serious threat to this species’ wild population in the near future. The most significant natural threats are long-term droughts, infrequent and irregular precipitation throughout the year, and habitat fragmentation. It has been recorded that there is massive grazing pressure on *P. sinaica*, especially by camels and donkeys [32, 69, 121].

#### Model performance evaluation for species distribution prediction

Evaluating model performance and acknowledging their limitations, including data uncertainty (from input errors or biases), model uncertainty (due to architectural choices and assumptions), parameter uncertainty (from estimation variability), and external factors (unpredictable environmental influences) is essential to prevent misinterpretation of results and ensure reliable habitat prioritization for conservation planning [123, 124]. Plant species exhibit varying responses to climate change based on their physiological and phenological traits [125], with observed impacts including shifts and alterations in species distributions [126]. To improve accuracy, ensemble modeling is preferred over single-model approaches for assessing climate-driven range shifts, as it reduces uncertainty, enhances robustness, and mitigates overfitting [127–131].

In addition, this study considered key environmental variables such as climate, topography, and soil properties, but model accuracy could be improved by incorporating additional factors like urbanization, biotic interactions, dispersal mechanisms, land cover changes, and population demographics, warranting further ecological and conservation research. Consequently, including more relevant non-climatic variables can enhance model accuracy and fine-tune model predictions, yielding a more realistic distribution prediction that captures the significance of the complex interaction between climate and geophysical factors [31, 132]. Although numerous field studies have examined endemic plant species in Egypt, critical knowledge gaps remain regarding the spatial distribution of threatened species, their ecological and population dynamics, habitat shifts, major threats, and conservation needs. The scarcity of detailed and systematic data limits the development and execution of robust conservation measures, both within and beyond protected areas.

Moreover, key challenge in species distribution modeling (SDM) involves balancing spatial resolution and sample size limitations. This study utilized environmental variables at a 1 km² (30 arc-second) resolution, recognizing that this relatively coarse scale may reduce prediction accuracy, especially in environmentally complex areas. However, this resolution was chosen due to data availability constraints, as consistent global-scale climate datasets at higher resolutions remain scarce. Notably, the 1 km² resolution has been widely adopted in SDM research, reflecting a practical compromise between precision and data accessibility. A critical challenge in species distribution modeling (SDMs) is the risk of overfitting, where models become overly tailored to training data, compromising their ability to generalize to new datasets. To mitigate this issue, we implemented several precautionary measures such as reducing spatial autocorrelation in occurrence records to minimize noise, selecting non-collinear environmental predictors, employing data partitioning and cross-validation during model calibration, simplifying model architecture, and utilizing ensemble techniques, all established best practices in SDM research [133, 134]. These strategies collectively enhanced model robustness, enabling our final ensemble model to demonstrate strong predictive performance on both test datasets and novel environmental scenarios.

Species distribution is determined by multiple factors, including physical, chemical, and biological aspects [135]. The geographic distribution of plants and animals can be influenced by factors such as soil temperature, moisture levels, and nutrient availability [136]. Therefore, identifying differences in the distribution of plant species is crucial to gaining a rational understanding of the environmental conditions needed for effective ecological restoration [137]. Species that are found in small, restricted areas where environmental factors can modify their suitability are the most threatened. Therefore, programs for ex situ conservation of endemic species are therefore emerging [138]. Aspect, Altitude and slope degree influence the microhabitats in Saint Catherine, which in turn influence the vegetation’s patterns and spatial distribution [139]. Models of species distribution measure the functional niche of a species at various spatial scales. In climate change scenarios, the analysis and findings can serve as spatial templates for addressing the viability of population, a species’ declining range or habitat, and natural sites for being introduced. Understanding the long-term survival of an endangered species in potential future climate scenarios can be obtained through SDM prediction [140].

Our results indicated that the models utilized in this study are helpful in locating appropriate habitats for the three plant species distributed in Saint Catherine. The present and future distribution of these species was predicted by the models with accuracy. Furthermore, these models demonstrated how elevational shifts and range expansion/contraction specific to a species will result from climate change. In this study, we utilized ensemble modeling approaches that include several algorithms as well as single algorithm approaches. The MaxEnt model provides excellent sample data availability, fast operation, and predictive power. Although it is typically utilized at the species level, it can also be used to investigate the range of distribution of community sets and plant functional types [141]. An ensemble modelling strategy integrating four algorithms was compared to the predicted capabilities of single-algorithm methods. Ensemble achieved high predictive performances based on AUC and TSS. Ensemble predicted maps show a high degree of similarity in terms of habitat appropriateness for current as well as future emission scenarios. According to some research [142, 143], Maxent is among the most dependable methods for simulating species distributions using insufficient data. On the other hand, according to Guillera-Arroita et al. [144], presence-absence methods, like GLM, can provide accurate predictions with small sample sizes if they are correctly assessed. They further stress that presence-absence is a more dependable method because it relies on the indications of species absence instead of random background points. Depending on the regional environment, climate factors have varying effects on species’ geographic distribution, which reflects the MaxEnt model’s sensitivity and accuracy to the input parameters [145]. Based only on simple bioclimatic variables, conclusions about the distribution of these species in the present and the future may be biased [112]. Therefore, we experimented with different variable combinations to improve our predictions [146]. This study incorporates edaphic variables along with climatic and topographic variables. This study elucidated that edaphic variables (cation exchange capacity, bulk density and organic carbon stocks, PH), climatic variables (wind) and topographic variables slope may be regarded as limitation variables for the potential geographic distribution of the four plant species in Saint Catherine. This result can be reinforced by that in mountainous areas, vegetation reacts to minute variations in the topography, such as slope, which have an impact on microclimatic conditions including soil moisture and temperature [147]. Moreover, slope topography, and vegetation composition of Saint Cathrine may have an impact on the variation in soil prosperities [148]. Because of the influence of bedrock, soils located on steeper slopes are typically less wet and less acidic [149]. These findings agree with [150]who predicted the distribution of 115 plant species in the western Alps of Canton deVaud, Switzerland using topo-climatic variables with edaphic variables and found that the three types of variables affect the distribution of plants. Additionally, 23 of the 25 rarest species from Mount Kaala, a narrow-endemism hotspot in New Caledonia, were modeled by [151] to see how their possible current distribution would change. He revealed that these kinds of variables affected the species’ distribution.

#### Current and future predictions

Our models showed that wind, Bio9, Bio3, water 10 and elevation were the most effective variables for *A. forskaohlii* subsp. *pubescens*; wind, Bio3, Bio15, clay, and elevation for *O. syriacum* subsp. *sinaicum*; wind, Bio3, Bio8, clay, aridity index and elevation for *P. sinaica* var. *sinaica*. These results align with established ecological understanding that precipitation serves as a key determinant of plant species distribution in mountain ecosystems, particularly acting as a dispersal constraint in arid environments [152, 153]. While elevation showed relatively lower contribution values in our models, we maintain its fundamental importance in species distribution modeling (SDMs) for mountainous regions. This perspective is supported by multiple studies demonstrating that elevation, when combined with climatic variables, significantly influences the spatial patterns of numerous plant species [64, 112]. It is clear that wind is the most contributed parameter in the distribution of the three studied taxa. In the Sinai Mountains, where pronounced topographic variation and diverse microhabitats exist, wind may play a significant role in shaping the potential distribution of endemic plant species. Specifically, wind can contribute to the formation of localized microclimatic conditions, influence soil moisture availability through enhanced evaporation rates and facilitate seed dispersal, particularly for species adapted to anemochory (wind dispersal) [154, 155]. Another study by Moustafa and Zaghloul [156] emphasized that elevation, soil characteristics, slope degree, and wind speed are the most significant factors controlling the distribution of plant communities in Catherine. DCA and first axis of CCA reflect the importance of the effect of the speed of summer winds where the sites which are exposed to the highest wind speeds are also the driest. This explain the low occurrence of the three studied taxa at high wind speed.

The potential existing appropriate habitats of *O. syriacum* subsp. *sinaicum* were indicated by the prediction of its distribution in El-Zawatin, W. Elarbain, W. Elfaraa, W. telah, W. El-talaa, and Farsh El-Romana in Saint Cathrine. Moustafa et al. [157] and Badawy et al. [158] mentioned the occurrence of this species in the same locations predicted by the models in this study. This species was found in a wide range of elevations between 1193 and 2051 m and in slope features between 88 and 89.9 degrees with acid, neutral, and alkaline soils with pH values ranging from 6 to 8.36. It also occupied the majority of high altitude representative different habitats in Saint Cathrine, such as wadi beds, terraces, gorges, slopes, and farshes [159]. Our prediction showed that wind, Bio3, Bio15, clay, and elevation were the most important variables explaining the potential distribution of *O. syriacum*. This is partially agrees with Mansour [159] who reported that *Origanum syriacum* subsp. *sinaicum’*s presence, growth, and distribution pattern are restricted by the micro-topography and micro-climate, which involves the orientation of sun exposure, the degree of mountain slopes, and the aspect degree. At broad spatial scales, Abiotic variables (soil, climate, and topography), energy availability, habitat area, the past and random events, recruitment restrictions, and immigration dynamics related to extinction have all been identified as significant contributors to the explanation of species richness [160].

Under the two scenarios SSP126 and SSP585 for the periods of 2041–2060 and 2061–2080, there will be a decline in the suitable habitat for this species. The number of mature individuals in the field is declining. This loss is caused by climate change and a protracted drought, which both decrease the quality of this species’ habitat. Climate change poses tremendous dangers to ecosystems around the world, and mountainous places with uncommon ecosystems, distinct landscapes, a huge number of endemic species, and enormous plant biodiversity are particularly vulnerable to the effects of climate change. Furthermore, climate change is accelerating plant invasion by reducing climatic barriers, jeopardizing the diversity of local plant species. Additionally, climate warming is causing habitat fragmentation and destruction, thereby intensifying the related consequences. The effects of climate change can change the makeup, arrangement, and operations of untouched mountain ecosystems, resulting in permanent losses of biodiversity [161]. Rising global temperatures are leading to changes in precipitation patterns, increased frequency of extreme weather events, and altered ecosystems in this region. These variables are affecting plant species distribution and abundance, perhaps causing shifts in ecosystem composition. Rapid environmental changes may pose challenges for native plants suited to specific climatic regimes, perhaps leading to the emergence of invasive species and new communities [162].

In addition, our results agrees with Parmesan and Yohe [163] who performed meta-analyses on over 1,700 species, fluctuations in range averaging 6.1 m per decade upward are in fact being caused by climate change. Lenoir et al. [164] evaluate changes in the optimal elevation of 171 forest plant species over six mountain ranges in France between 1905 and 1985 and 1986–2005 and demonstrate evidence of upslope migration of plant species in the montane belt, with an average shift of 29.4 m per decade, two thirds of the plant species under study exhibited an upward shift. According to their findings, there has been an upward movement in both the upper and lower distributional margins, indicating that species are impacted by climate change throughout their whole range, not only at the boundaries. Certainly, there have been documented worldwide tendencies towards increasing range limit changes and shifting community compositions on tops of mountains, which are frequently linked to climate change [165–167]. The reason for this can be attributed to various factors such as temperature variations, precipitation patterns, water balance, bare soil surface area, increased atmospheric carbon dioxide levels, and plant range shifts [168–170].

Moreover, our results align with the findings of Omar and Elgamal [171], who anticipated such a decrease in *Micromeria serbaliana* within SKP. In addition, El-Khalafy et al. [172] predicted that that distribution range of *Micromeria serbaliana* would decline between 2061 and 2080. Furthermore, Abdelaal et al. [31] et al. forecasted that the habitat suitability for *Primula boveana* would diminish because of future global warming projected for the years 2050 and 2070. Serag et al. [173] stated that on the long run and with harsher climate change scenarios, there could be a severe decline in the Population of mountainous taxa *Phlomis aurea*. Additionally, recent studies conducted in the same area corroborated the observed shifts in the geographic distribution of the native *Rosa arabica* species [174]. Extended drought, sudden flooding leading to uprooting and overgrazing will contribute to the loss of part of the habitat, which will affect the size, cover, sensitivity, vitality, and distribution of the species. As a result, the habitat for these two species will become fragmented [69].

In addition, the models in this study showed that the current suitable habitat for *A. forskaohlii* subsp. *pubescens* is located in in Wadi Gebal, AL-Gebel Al-Ahmar, Wadi Abu tweita and Wadi Al-Arbain. This species was found in the same habitats predicted by the models in this study [66, 175]. The best growth conditions for this species are stony soils at the tops of mountains, gravelly soils in wadis and plains, and sandy, alkaline and nonsaline to slightly saline soils. It is restricted to gorges and slopes [152]. Our prediction showed that variables that have the greatest significance in explaining the possible distribution of *A. forskaohlii* subsp. *pubescens* were wind, Bio9, Bio3, water 10 and elevation.

Moreover, the area of the presently suitable localities for *P. sinaica* is located in El-Zawatin, W. Elarbain, W. Elfaraa, W. Telah, W. El-Talaa, and Farsh El-romana. Omaretal. [69] stated that this species was found in the same habitats predicted by this study and is located at cliffs and gorges with sharp slopes, low temperature and rainfall and sandy to loamy sand, alkaline soil. Our prediction showed that variables that have the greatest significance in explaining the possible distribution of *P. sinaica* were wind, Bio3, Bio8, clay, aridity index and elevation. Omar et al. [176] predicted that the Precipitation and temperature have the high contributions for limiting the distribution of this species. Moreover, Jiang et al. [177] found that precipitation, temperature, and altitude were the main variables that affected the distribution of *Polygala tenuifolia* in China.

Between 2041 and 2060 and 2061–2080, and under the two scenarios SSP126 and SSP585, the suitable habitats for *A. forskaohlii* subsp. *pubescens* and *P. sinaica* var. *sinaica* will increase. Our findings agree with chen et al. [178] and Wilson et al. [179] that reported that in response to climatic changes, animal and plant species have shown recent alterations in both latitudinal and altitudinal distributions, with ranges growing at high latitudes and altitudes and shrinking at lower latitudes and altitudes. This can be attributed to the fact that altitudinal alterations are primarily caused by changes in the temperature regime and availability of water, with negative changes in both producing the highest pressure [165]. In addition, similar results were recorded by Refaat et al. [180] who predicted a notable expansion in the current suitable area of the *Silene schimperiana* that is endemic to St. Catherine as well in both the 2050 and 2070 scenarios. Moreover, there is evidence reported by Moustafa and Zayed [152] that plants that live in mountainous areas in microhabitats typically like rocky outcrops, slopes, terraces, gorges, wadi beds and other well-drained areas are expected to have a greater capacity to adapt to climate change than those with narrow ecological niches. Notably, in St. Catherine, where high elevations can doubtlessly work as ecological barriers that restrict distributions of some species, but also, it’s a well-protected area where the Egyptian Environmental Agency team induced conservation actions can surely help other species expanding new habitats in the future.

Moreover, the target species, responding to climate change pressures, is projected to shift toward higher elevation refugia, particularly in mountainous areas that mirror its current elevational range. However, this climate adaptation strategy faces major constraints due to the fragmented and isolated nature of suitable high-altitude habitats [181, 182]. The predicted habitat losses, appearing as small, dispersed patches in peripheral mountain zones, underscore this limitation - the species’ capacity to colonize these newly suitable but disconnected areas remains doubtful and requires urgent field validation and long-term monitoring.

These results corroborate extensive research demonstrating climate change’s profound influence on species redistribution. Multiple studies confirm that mountain-dwelling species are being forced to migrate upward along elevational gradients [183, 184]. For example, Manish et al. [184] project that 17–18% of Himalayan endemics could lose their suitable habitats by 2050–2070, triggering range shifts where generalist species ascend and intensify competition pressure on summit-restricted endemics. Similarly, Di Musciano et al. [185] documented how upward-shifting lowland species threaten rare high-elevation specialists in the Apuan Alps, highlighting the complex ecological cascades triggered by climate-mediated range shifts.

## Conclusion

The study explored the current distribution and predicted habitat suitability of three endemic plant species in SKP diverse ecosystems under various climate change scenarios using SDMs. Results demonstrated the intricate relationships between habitat suitability and environmental variables, highlighting the importance of factors like climatic parameters and elevation. Our research specifically showed that wind and climate variables were dominant in shaping the potential distribution of the three taxa. It is important to highlight that the decrease in biodiversity due to projected climate conditions emphasizes the pressing requirement for conservation initiatives, particularly in light of the risks presented by human actions like alterations in land use and the fragmentation of habitats. Ensuring the safety of these species by managing the risks they face and putting protection measures in place is crucial. It is important to establish and enforce laws and regulations to guarantee their protection. Our findings emphasize the importance of conservation actions like reintroduction, in situ and *ex situ* conservation planning in suitable environments.

## Data Availability

No datasets were generated or analysed during the current study.
